# Identifying the relative contributions of body size across life course to midlife and late-life cognitive function: a Bayesian analysis from the Guangzhou Biobank Cohort Study

**DOI:** 10.1016/j.jnha.2026.100799

**Published:** 2026-01-30

**Authors:** Sihan Hou, Jiao Wang, Tai Hing Lam, Kar Keung Cheng, Wei Sen Zhang, Lin Xu

**Affiliations:** a School of Public Health, Sun Yat-sen University, Guangzhou 510080, China; b Guangzhou Twelfth People’s Hospital, Guangzhou 510620, China; c School of Public Health, the University of Hong Kong, Hong Kong SAR, China; d Institute of Applied Health Research, University of Birmingham, Birmingham, United Kingdom

**Keywords:** Body size, Obesity, Cognitive function, Life-course perspective, Bayesian relevant life course exposure model

## Abstract

**Objectives:**

The relative contributions of life-course obesity to midlife and late-life cognitive function have not been reported. We examined the association of life-course body size with cognitive function and identified the relative contribution of body size at each life stage.

**Design:**

This was a study based on data from the Guangzhou Biobank Cohort Study.

**Setting:**

A community-based population in China.

**Participants:**

9,303 participants without a history of dementia or other serious mental disorders were included, with a mean age of 59.9 years (standard deviation = 6.0 years).

**Measurements:**

Perceived childhood, adolescence, early adulthood, midlife, and current body size were assessed by Stunkard’s Figure Rating Scale (labelled 1–9, from very thin to very fat), and analysed as categorical or continuous variables. Cognitive function was assessed using the Mini-Mental State Examination (MMSE), and analysed as a continuous score. A Bayesian relevant life course exposure model was used to quantify the relative contributions of body size to cognitive function.

**Results:**

After adjustment for confounders, each one-figure increase in body size was associated with lower MMSE scores. The βs (95% confidence intervals) were −1.121 (−1.200, −1.043), −1.077 (−1.161, −0.993), −0.795 (−0.871, −0.719), −0.450 (−0.520, −0.380), −0.253 (−0.318, −0.188) for childhood, adolescence, early adulthood, midlife and current status, respectively. The contributions of larger body size to poorer cognitive function varied across life stages, with childhood and adolescence accounting for 58.96% (95% credible interval (CrI) = 49.81%–68.07%) and 38.52% (95% CrI = 29.11%–47.82%) of the association, respectively.

**Conclusion:**

Body size in childhood and adolescence mainly explained the negative association between life-course body size and cognitive function in midlife and late-life. This finding highlights the importance of early-life obesity prevention for maintaining cognitive function.

## Highlights

• Larger body size at each life stage was associated with poorer cognitive function.
• Associations were observed in orientation, attention and calculation, and language.
• Childhood and adolescence were sensitive stages for maintaining cognitive function.

## Graphical abstract

**Figure:**
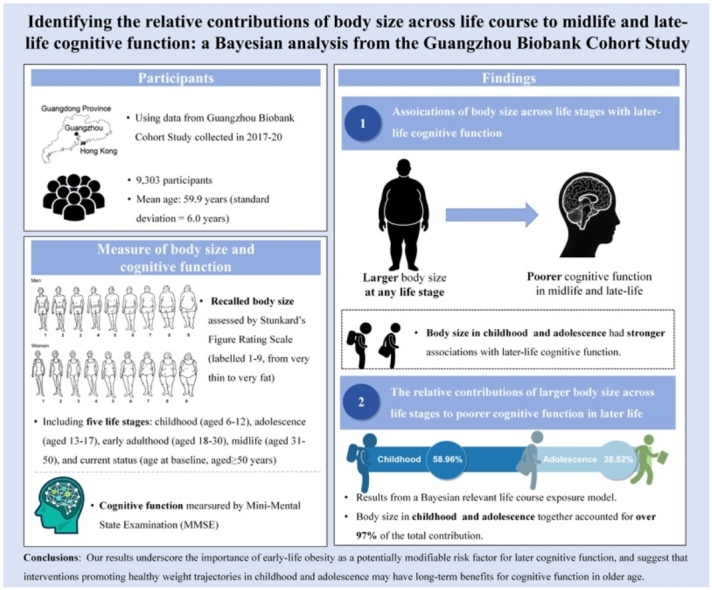

## 1 Introduction

Cognitive impairment and dementia have become pressing public health challenges worldwide due to rapidly aging populations [1,2], leading to substantial health burden [3]. Identifying modifiable risk factors across life course is needed for developing effective preventive strategies.

A growing body of evidence suggests that midlife obesity has been associated with a higher risk of dementia [4]. However, dementia is characterized by a prolonged preclinical period [5], highlighting the importance of considering risk factors in earlier life stages for neurodegenerative diseases.

Furthermore, childhood and adolescence are the critical periods of neurodevelopment [6]. The global epidemic of obesity in these age groups [7] has raised great concern regarding its short-term consequence on brain structure [8] and cognitive function [9]. However, the long-term cognitive implications of early-life obesity remain unclear. A limited number of studies, mostly in White and Black populations, have examined body mass index (BMI) during adolescence and early adulthood in relation to midlife cognitive function, but the findings were inconsistent [[10], [11], [12], [13], [14], [15], [16]]. Some studies included only measurement at a single early-life stage [[10], [11], [12],^16^]. This gap largely reflects the challenge of conducting longitudinal studies with objectively measured BMI beginning in early life. Additionally, retrospective recall of numerical BMI values from early decades is often unreliable. Therefore, the figure rating scale offers a feasible alternative for capturing perceived body size across the life course. It was previously used in a Brazilian longitudinal study to evaluate the association of recalled body size trajectory with midlife and late-life cognitive decline [17]. However, no study to date has reported the relative contributions of life-course body size to midlife and late-life cognitive function.

To address these gaps, we used data from Guangzhou Biobank Cohort Study (GBCS) to examine the association between life-course body size from childhood and cognitive function in midlife and late-life, and further identify the relative contribution of body size at each life stage.

## 2 Material and methods

### 2.1 Study population

This is a retrospective study. We used GBCS data collected from 2017 to 2020. Participants were included in GBCS if they were permanent Guangzhou residents and without a history of dementia or other serious mental disorders that could interfere with cognitive assessment [18]. Ethics approval was granted by the Guangzhou Medical Ethics Committee of the Chinese Medical Association, and all participants provided written, informed consent.

### 2.2 Body size across life stages

Perceived body size was assessed using Stunkard’s Figure Rating Scale [19], a validated proxy measure of weight status in the absence of objectively measured BMI [20]. The scale consists of nine sex-specific silhouettes ranging from extreme thinness (labelled 1) to extreme obesity (labelled 9) (Figure S1). At baseline, participants were asked to retrospectively select the silhouette that best represented their body size during five distinct life stages: childhood (6−12 years), adolescence (13−17 years), early adulthood (18−30 years), midlife (31−50 years), and current status (age at baseline, aged ≥50 years) (Figure S2).

### 2.3 Cognitive function

Cognitive function was assessed by Mini-Mental State Examination (MMSE), including orientation to time and place, registration, attention and calculation, recall, and language and praxis domains [21]. Higher MMSE scores indicate better cognitive function.

### 2.4 Potential confounders

Given the established associations between both childhood and adulthood socioeconomic position and both obesity and cognitive function [22], we included sex, age, childhood socio-economic disadvantage (CSD), and adulthood social-economic characteristics (education, occupation, and family annual income) as potential confounders. CSD was assessed using a composite score derived from two domains: parental material possessions and indicators of material deprivation during childhood [23]. Higher CSD scores indicate worse childhood socioeconomic position. Detailed definitions of all confounders were provided in the Supplemental Methods.

### 2.5 Statistical analysis

We used linear regression model to analyse association between body size and cognitive function, yielding regression coefficients (*β*s) and 95% confidence intervals (95% CIs). We also did analyses stratified by sex or age, and examined whether the interaction was statistically significant using likelihood ratio test. R software (version 4.5.1) was used for data analyses. All tests were two-sided with a significant level of *P* < 0.05.

To quantify the relative contributions of body size at different life stages to cognitive function, we used Bayesian relevant life course exposure model (BRLM), as proposed by Madathil et al. [24]. The relative contributions (i.e., weights) of body size at different life stages and the 95% credible intervals (CrIs) were estimated by Bayesian inference. Weights were constrained to lie between 0 and 1, with the sum equal to 1. Details of BRLM were provided in the Supplemental Methods.

In the sensitivity analyses, we further adjusted for current BMI (n = 9,197). Additionally, participants with an MMSE recall-domain score <2 were excluded to minimize potential recall error regarding retrospective body size assessment (n = 8,099).

## 3 Results

### 3.1 Baseline characteristics

Of 10,514 participants at baseline, we excluded participants with missing data on body size across life stages (N = 187), cognitive function (N = 74) and potential confounders (N = 950). Thus, a total of 9,303 participants were included in the main analyses.

Table 1 shows that the mean age of participants was 59.9 years (standard deviation (SD) = 6.0), 71.5% were women. 9.7% had an education level of primary or below. 25.3% were manual workers, and 32.6% had a family annual income lower than 50,000. The mean (SD) of CSD and total MMSE scores were 2.3 (1.9) and 27.2 (3.9), respectively.

**Table 1:** Characteristics of participants in Guangzhou Biobank Cohort Study.

	Total (N = 9,303)
Age (years), mean (SD)	59.9 (6.0)
Sex, N (%)	
Women	6,656 (71.5)
Men	2,647 (28.5)
Education, N (%)	
Primary school or below	903 (9.7)
Junior middle school	1,909 (20.5)
Senior middle school	4,766 (51.2)
College or above	1,725 (18.5)
Occupation, N (%)	
Manual	2,354 (25.3)
Non-manual	1,596 (17.2)
Others	5,353 (57.5)
Family annual income, CNY/year, N (%)	
<50,000	3,033 (32.6)
50,000−79,999	3,415 (36.7)
≥80,000	2,755 (29.6)
Don't know	100 (1.1)
Childhood socio-economic disadvantage, mean (SD)	2.3 (1.9)
Mini-Mental State Examination scores, mean (SD)	
Total score	27.2 (3.9)
Orientation-domain score	9.3 (1.6)
Registration-domain score	2.8 (0.5)
Attention and calculation-domain score	4.1 (1.1)
Recall-domain score	2.4 (0.8)
Language and praxis-domain score	8.6 (0.9)

CNY: Chinese yuan, SD: standard deviation.

### 3.2 Association between body size at each life stage and cognitive function

After adjusting for sex, age, education, occupation, family annual income and CSD, participants selecting the fifth figure or above showed significantly lower MMSE scores compared to those selecting the fourth figure during childhood, adolescence, early adulthood, midlife and current status (Table 2). When body size was treated as a continuous variable, each one-figure increase in body size was associated with lower MMSE scores. The magnitude of these associations was greatest for childhood (β = −1.121, 95% CI −1.200, −1.043) and adolescence (β = −1.077, 95% CI −1.161, −0.993), and attenuated in early adulthood (β = −0.795, 95% CI −0.871, −0.719), midlife (β = −0.450, 95% CI −0.520, −0.380) and current status (β = −0.253, 95% CI −0.318, −0.188).

**Table 2:** Association between body size at each life stage and MMSE scores.

	N (%)	Crude model, β (95% CI)	Model 1, β (95% CI) †	Model 2, β (95% CI) ‡
Childhood (6−12 years)				
≤3 (smallest figures)	1016 (10.9)	−0.262 (−0.509, −0.016) *	−0.223 (−0.470, 0.024)	0.114 (−0.113, 0.341)
4	4949 (53.2)	0.000 (ref)	0.000 (ref)	0.000 (ref)
5	1849 (19.9)	−2.437 (−2.631, −2.242) ***	−2.456 (−2.651, −2.261) ***	−2.043 (−2.229, −1.858) ***
≥6 (largest figures)	1489 (16.0)	−3.562 (−3.773, −3.351) ***	−3.581 (−3.792, −3.370) ***	−2.788 (−2.992, −2.585) ***
* P*_trend_		<0.001	<0.001	<0.001
Per 1-figure increase		−1.350 (−1.430, −1.270) ***	−1.370 (−1.451, −1.289) ***	−1.121 (−1.200, −1.043) ***
Adolescence (13−17 years)				
≤3 (smallest figures)	554 (6.0)	−0.424 (−0.748, −0.100) *	−0.381 (−0.706, −0.057) *	0.101 (−0.197, 0.398)
4	4830 (51.9)	0.000 (ref)	0.000 (ref)	0.000 (ref)
5	2396 (25.8)	−1.984 (−2.165, −1.804) ***	−2.011 (−2.192, −1.830) ***	−1.454 (−1.626, −1.282) ***
≥6 (largest figures)	1523 (16.4)	−3.476 (−3.688, −3.264) ***	−3.500 (−3.712, −3.287) ***	−2.604 (−2.810, −2.399) ***
* P*_trend_		<0.001	<0.001	<0.001
Per 1-figure increase		−1.381 (−1.467, −1.296) ***	−1.402 (−1.488, −1.316) ***	−1.077 (−1.161, −0.993) ***
Early adulthood (18−30 years)				
≤3 (smallest figures)	368 (4.0)	−0.312 (−0.714, 0.091)	−0.273 (−0.676, 0.130)	0.249 (−0.118, 0.616)
4	3578 (38.5)	0.000 (ref)	0.000 (ref)	0.000 (ref)
5	3674 (39.5)	−1.171 (−1.344, −0.998) ***	−1.196 (−1.370, −1.023) ***	−0.868 (−1.028, −0.708) ***
≥6 (largest figures)	1683 (18.1)	−3.148 (−3.365, −2.930) ***	−3.187 (−3.405, −2.968) ***	−2.229 (−2.438, −2.020) ***
* P*_trend_		<0.001	<0.001	<0.001
Per 1-figure increase		−1.025 (−1.104, −0.946) ***	−1.042 (−1.122, −0.962) ***	−0.795 (−0.871, −0.719) ***
Midlife (31−50 years)				
≤3 (smallest figures)	221 (2.4)	−0.457 (−0.984, 0.070)	−0.429 (−0.956, 0.099)	−0.120 (−0.596, 0.355)
4	2629 (28.3)	0.000 (ref)	0.000 (ref)	0.000 (ref)
5	3343 (35.9)	−1.246 (−1.442, −1.050) ***	−1.254 (−1.450, −1.057) ***	−0.725 (−0.906, −0.543) ***
≥6 (largest figures)	3110 (33.4)	−1.935 (−2.134, −1.735) ***	−1.961 (−2.161, −1.760) ***	−1.177 (−1.370, −0.984) ***
* P*_trend_		<0.001	<0.001	<0.001
Per 1-figure increase		−0.728 (−0.801, −0.655) ***	−0.739 (−0.812, −0.666) ***	−0.450 (−0.520, −0.380) ***
Current status (age at baseline, aged ≥50 years)				
≤3 (smallest figures)	188 (2.0)	−0.629 (−1.201, −0.057) *	−0.607 (−1.179, −0.034) *	−0.325 (−0.840, 0.189)
4	2389 (25.7)	0.000 (ref)	0.000 (ref)	0.000 (ref)
5	2454 (26.4)	−1.911 (−2.128, −1.694) ***	−1.922 (−2.139, −1.704) ***	−0.949 (−1.149, −0.749) ***
≥6 (largest figures)	4272 (45.9)	−1.281 (−1.473, −1.088) ***	−1.296 (−1.490, −1.102) ***	−0.727 (−0.915, −0.539) ***
* P*_trend_		<0.001	<0.001	<0.001
Per 1-figure increase		−0.358 (−0.426, −0.290) ***	−0.363 (−0.431, −0.294) ***	−0.253 (−0.318, −0.188) ***

CI: confidence interval.

*P < 0.05, ***P < 0.001.

† Model 1 were adjusted for sex, age.

‡ Model 2 were additionally adjusted for education, occupation, family annual income and childhood socio-economic disadvantage (CSD).

Large body size was inversely associated with orientation, attention and calculation, and language and praxis domains across all life stages, but not with registration or recall domain (Table S1-S5). A clear dose-response pattern was observed in the orientation domain, particularly for body size in childhood through midlife (Figure S3), and similar trends were found when body size was treated as a continuous variable (Figure S4).

In sex-stratified analyses, a statistically significant interaction was only found between sex and current body size (as categorical variable) (*P* for interaction = 0.01, Figure S5 & Figure S6). Women selecting the fifth figure and the sixth figure or above had similar associations with MMSE scores. In contrast, men selecting the fifth figure had the lowest MMSE scores, followed by those selecting the sixth figure or above. Statistically significant interactions between age and body size across all life stages were identified (all *P* for interaction <0.05, Figure S7 & Figure S8). Compared to participants with age ≥65, those with age <65 had stronger negative associations between body size and MMSE scores.

### 3.3 The relative contribution of body size at each life stage

**Fig. 1:**
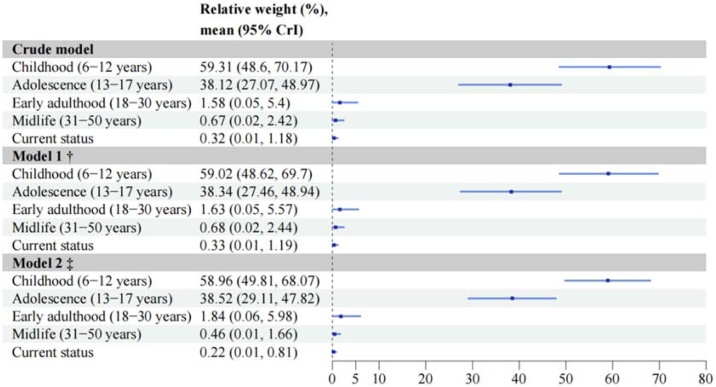
Posterior mean weight and their 95% credible intervals of body size at each life stage on midlife and late-life cognitive function. ^†^Model 1 adjusted for sex, age. ‡Model 2 additionally adjusted for education, occupation, family annual income and childhood socio-economic disadvantage (CSD). CrI: credible interval

After adjusting for sex, age, education, occupation, family annual income and CSD, body size in childhood and adolescence predominantly explained the negative association of life-course body size with later-life cognitive function (Fig. 1 & Figure S9). The posterior mean weights for body size in childhood and adolescence were 58.96% (95% CrI 49.81%, 68.07%) and 38.52% (95% CrI 29.11%, 47.82%), respectively. The sensitive period hypothesis was supported as the best-fitting life course model, based on the shortest Euclidean distance between observed and expected weight distributions (Figure S10). The sex or age-specific analyses also showed similar results (Table S6 & Table S7).

### 3.4 Sensitivity analyses

Similar results were observed after further adjusting for current BMI (Figure S12-S17 & Table S8-S11) or excluding participants with an MMSE recall-domain score <2 (Figure S18-S23 & Table S12-S15).

## 4 Discussion

In this large population-based study, we found that larger body size across all life stages was significantly associated with poorer cognitive function in later life. Notably, using the BRLM, we first found that body size in childhood and adolescence together explained over 97% of the observed association of life-course body size with later-life cognitive function. Our results suggest that interventions targeting early-life obesity may have long-term benefits for cognitive function.

Our results were generally consistent with previous studies, showing obesity in early life by recalled body size was associated with poor cognitive function [17,25], but further identified the relative contributions of life-course body size to cognitive function in later life. The Brazilian cohort study found that compared with stable normal weight, those with weight gain or persistent overweight trajectories from 5 to 40 years were associated with faster cognitive decline [17]. However, as this Brazilian study enrolled participants from public universities, the findings were mainly restricted to those with high education levels [17]. By contrast, our community-based study included participants with different education levels, reducing potential selection bias. In the UK Biobank study, self-reported retrospectively plumper body size at age 10, compared to the average body size of peers, was associated with a higher risk of dementia over 8.7 years of follow-up in participants without dementia at baseline [25]. However, this assessment relied on a single subjective question and did not include adolescence or early adulthood body size [25], in contrast to the figure-based, multi-stage assessments used in our study.

However, some studies used self-reported retrospectively BMI found no association between early adulthood obesity and incident dementia risk [10], or late-midlife cognitive function [11]. As these studies focused only on early adulthood [10,11], results could not be directly compared with ours, which quantified the contributions of body size across life stages.

Mechanisms underlying the implication of early-life obesity on long-term cognitive function can be explained by animal and human evidence. In a murine model, early-life high-fat diet induced irreversible epigenetic modifications, leading to synaptic dysfunction and proinflammatory responses later in life, even after dietary normalization [26]. In human, childhood obesity frequently tracks into adulthood [27], and prolonged obesity is associated with reduced adiponectin and elevated proinflammatory adipokines, which may promote β-amyloid accumulation and structural brain changes, including alterations in white-matter volume [28].

The strengths in our study included the life-course perspective beginning in childhood and the application of BRLM to quantify relative contributions of body size. However, several limitations should be noted. First, body size was self-reported retrospectively using a figure rating scale, which, although more feasible than recalling numerical BMI, particularly in childhood, might be subject to recall and misclassification bias. Second, residual confounders such as APOE genotype and childhood cognitive ability might exist. Third, cognitive function was assessed only using MMSE at a single time point. Further studies incorporating broader, repeated cognitive assessments and genetic data are needed to confirm and extend our findings. Fourth, although the BRLM is useful for quantifying the relative contribution of body size at each life stage, our findings cannot establish causal inference given the observational nature of this study.

## 5 Conclusions

Body size in childhood and adolescence mainly explained the negative association between life-course body size and later-life cognitive function. This finding highlights the importance of early-life obesity prevention as a beneficial strategy to midlife and late-life cognitive function.

## CRediT authorship contribution statement

Sihan Hou: Conceptualization, Methodology, Funding acquisition, Formal analysis, Visualization, Writing – original draft, Writing – review & editing. Jiao Wang: Conceptualization, Methodology, Funding acquisition. Tai Hing Lam: Conceptualization, Methodology, Funding acquisition, Writing – review & editing, Supervision. Weisen Zhang: Conceptualization, Methodology, Funding acquisition. Lin Xu: Conceptualization, Methodology, Funding acquisition, Writing – review & editing, Supervision.

## Ethics approval

The Guangzhou Medical Ethics Committee of the Chinese Medical Association approved the study.

## Declaration of Generative AI and AI-assisted technologies in the writing process

Authors did not use generative artificial intelligence (AI) and AI-assisted technologies in the writing process.

## Funding

This work was supported by the 10.13039/501100001809National Natural Science Foundation of China [grant numbers 82373661].

## Data availability

Due to ethical restrictions protecting patient privacy, data available on request from the Guangzhou Biobank Cohort Study Data Access Committee. Please contact us at gbcsdata@hku.hk for study protocol, statistical code and dataset from which the results were derived.

## Declaration of competing interest

The authors declare that they have no known competing financial interests or personal relationships that could have appeared to influence the work reported in this paper.
