# Supplementary material for: Identifying the relative contributions of body size across life course to midlife and late-life cognitive function: a Bayesian analysis from the Guangzhou Biobank Cohort Study
Source: J Nutr Health Aging. 2026 Jan 30;30(4):100799. doi: 10.1016/j.jnha.2026.100799 (PMC12878604; doi:10.1016/j.jnha.2026.100799)
Supplement: Supplementary file 1 [file mmc1.docx]

**Supplemental Methods**

***Potential confounders***

Childhood socio-economic disadvantage (CSD) consisted of parental material possessions and indicators of material deprivation during childhood [1]. Parental material possessions were assessed based on the cultural and historical characteristics in southern China in the mid-20th century, including three simple items (i.e., parental ownership of a bike, a sewing machine, and a watch). Each item was assigned as zero for the present or one for the absent. Childhood material deprivation was assessed by four items including experiences of childhood hunger, type of childhood residence, frequency of meat consumption during childhood, and whether new clothes were purchased during the Spring Festival. Each item was assigned as one when the answer was “Daily” hunger, “Rural” residence, “Never” consuming meat and “No” new clothing, and as zero otherwise. A summed CSD score was calculated by summing all seven items, yielding a range from 0 to 7.

Education was classified into four levels: primary school or below, junior middle school, senior middle school, and college or above. Occupation was categorised as manual (agricultural work, factory work, or sales and services), non-manual (administrative/managerial, professional/technical, or military/police), or others (housewife/husband or retired). Family annual income was grouped into four categories: <50,000 CNY/year, 50,000–79,999 CNY/year, ≥80,000 CNY/year, and unknown.

***Statistical analysis***

The Bayesian relevant life course exposure model (BRLM) proceeded in two stages:

$$\omega_{i}=\sum_{t=1}^{5} \omega_{t}*x_{ti}$$

$${(MMSE scores)}_{i}= \beta_{0}+ \delta* \omega_{i}+ \lambda* C_{i}$$

In the first equation, the *ω_t_* represented the relative contributions (i.e., weights) of body size at life stage *t* (from childhood to current status, *t* =1,2,3,4,5), estimated by Bayesian inference. *x_ti_* represented body size at time *t* for participant *i* (*i*=1, 2, ..., *N*), and the *ω_i_* represented the weighted sum of body size across all life stages. In the second equation, MMSE scores for participant i was regressed on *ω_i_*, with *δ* representing the total effect of cumulative life course exposure and *λ* being a vector of coefficients for *p* potential confounders (*C_i_* =*c*_1_*_i_*, *c*_2_*_i_*, …, *c*_p_*_i_*), including sex, age, CSD, adulthood social-economic position. As no prior information was available regarding the life course structure of effects, we used uninformative priors on weights for the five life stages: a Dirichlet prior distribution (1,1,1,1,1), and weakly informative Cauchy (0, 2.5) distributions for *β*_0_ and *δ*. Model estimation was performed using four parallel Hamiltonian Monte Carlo chains, with the first 10,000 iterations for burn-in and subsequent 20,000 iterations for inference. Convergence was assessed using trace plots (Figure S11) and R-hat statistics.

The BRLM enabled the data-driven identification of the life course hypothesis that best explained the associations between body size at five distinct life stages and midlife and late-life cognitive function. The BRLM accommodated three primary life course hypotheses: the accumulation hypothesis (equal contribution of exposure across all stages), the sensitive period hypothesis (differential contributions across stages), and critical period hypothesis (a single stage of predominant importance). To determine the most plausible life course model, we compared observed weights to those expected under theoretical life course scenarios: the accumulation model assumed equal weight across five stages (0.2 for each), critical model assumed the weight at one stage was 1 and the rest were 0, and the sensitive model assigned weights of 0.5 (childhood) and 0.125 for each subsequent stage. The best-fitting model was identified by the minimum Euclidean distance between observed and expected weights. The BRLM analyses were done using the “rstan” package in R [2].

***References***

[1] Huang YY, Zhang WS, Jiang CQ, Zhu F, Jin YL, Au Yeung SL, et al. Childhood socio-economic disadvantages versus adverse care experiences: Mediation and moderation impacts on late-life depressive symptoms. Eur Psychiatry. 2024;67:e47. 10.1192/j.eurpsy.2024.1760.

[2] Carpenter B, Gelman A, Hoffman MD, Lee D, Goodrich B, Betancourt M, et al. Stan: A Probabilistic Programming Language. J Stat Softw. 2017;76. 10.18637/jss.v076.i01.

**Supplemental Figures & Tables**

**Figure S1** Nine men or women body figures from Stunkard’s Figure Rating Scale

**Figure S2** Distributions of body size at different life stages

**Figure S3** Association between body size (as categorical variable) at each life stage with domain-specific cognitive function (summary of model 2)

**Figure S4** Association between body size (as continuous variable) at each life stage with domain-specific cognitive function (summary of model 2)

**Figure S5** Sex-specific associations between body size (as categorical variable) at each life stage with cognitive function

**Figure S6** Sex-specific associations between body size (as continuous variable) at each life stage with cognitive function

**Figure S7** Age-specific associations between body size (as categorical variable) at each life stage with cognitive function

**Figure S8** Age-specific associations between body size (as continuous variable) at each life stage with cognitive function

**Figure S9** Posterior distributions of relative weights across life stages on midlife and late-life cognitive function

**Figure S10** Posterior distributions of Euclidean distance between the expected and estimated weights of body size on midlife and late-life cognitive function

**Figure S11** Trace plots of the four parallel Hamiltonian Monte Carlo chains based on Bayesian relevant life course exposure model

**Figure S12** Association between body size (as categorical variable) at each life stage with domain-specific cognitive function further adjusting for current body mass index (summary of model 2)

**Figure S13** Association between body size (as continuous variable) at each life stage with domain-specific cognitive function further adjusting for current body mass index (summary of model 2)

**Figure S14** Sex-specific associations between body size (as categorical variable) at each life stage with cognitive function further adjusting for current body mass index

**Figure S15** Sex-specific associations between body size (as continuous variable) at each life stage with cognitive function further adjusting for current body mass index

**Figure S16** Age-specific associations between body size (as categorical variable) at each life stage with cognitive function further adjusting for current body mass index

**Figure S17** Age-specific associations between body size (as continuous variable) at each life stage with cognitive function further adjusting for current body mass index

**Figure S18** Association between body size (as categorical variable) at each life stage with domain-specific cognitive function after excluding those with an MMSE recall-domain score < 2 (summary of model 2)

**Figure S19** Association between body size (as continuous variable) at each life stage with domain-specific cognitive function after excluding those with an MMSE recall-domain score < 2 (summary of model 2)

**Figure S20** Sex-specific associations between body size (as categorical variable) at each life stage with cognitive function after excluding those with an MMSE recall-domain score < 2

**Figure S21** Sex-specific associations between body size (as continuous variable) at each life stage with cognitive function after excluding those with an MMSE recall-domain score < 2

**Figure S22** Age-specific associations between body size (as categorical variable) at each life stage with cognitive function after excluding those with an MMSE recall-domain score < 2

**Figure S23** Age-specific associations between body size (as continuous variable) at each life stage with cognitive function after excluding those with an MMSE recall-domain score < 2

**Table S1** Association between body size at each life stage with the orientation domain

**Table S2** Association between body size at each life stage with the registration domain

**Table S3** Association between body size at each life stage with the attention and calculation domain

**Table S4** Association between body size at each life stage with the recall-domain

**Table S5** Association between body size at each life stage with the language and praxis domain

**Table S6** Sex-specific posterior mean weights of body size across life stages and their 95% credible intervals on midlife and late-life cognitive function

**Table S7** Age-specific posterior mean weights of body size across life stages and their 95% credible intervals on midlife and late-life cognitive function

**Table S8** Association between body size at each life stage and MMSE scores further adjusting for current body mass index

**Table S9** Posterior mean weights of body size across life stages and their 95% credible intervals on midlife and late-life cognitive function further adjusting for current body mass index

**Table S10** Sex-specific posterior mean weights of body size across life stages and their 95% credible intervals on midlife and late-life cognitive function further adjusting for current body mass index

**Table S11** Age-specific posterior mean weights of body size across life stages and their 95% credible intervals on midlife and late-life cognitive function further adjusting for current body mass index

**Table S12** Association between body size at each life stage and MMSE scores after excluding those with an MMSE recall-domain score < 2

**Table S13** Posterior mean weights of body size across life stages and their 95% credible intervals on midlife and late-life cognitive function after excluding those with an MMSE recall-domain score < 2

**Table S14** Sex-specific posterior mean weights of body size across life stages and their 95% credible intervals on midlife and late-life cognitive function after excluding those with an MMSE recall-domain score < 2

**Table S15** Age-specific posterior mean weights of body size across life stages and their 95% credible intervals on midlife and late-life cognitive function after excluding those with an MMSE recall-domain score < 2


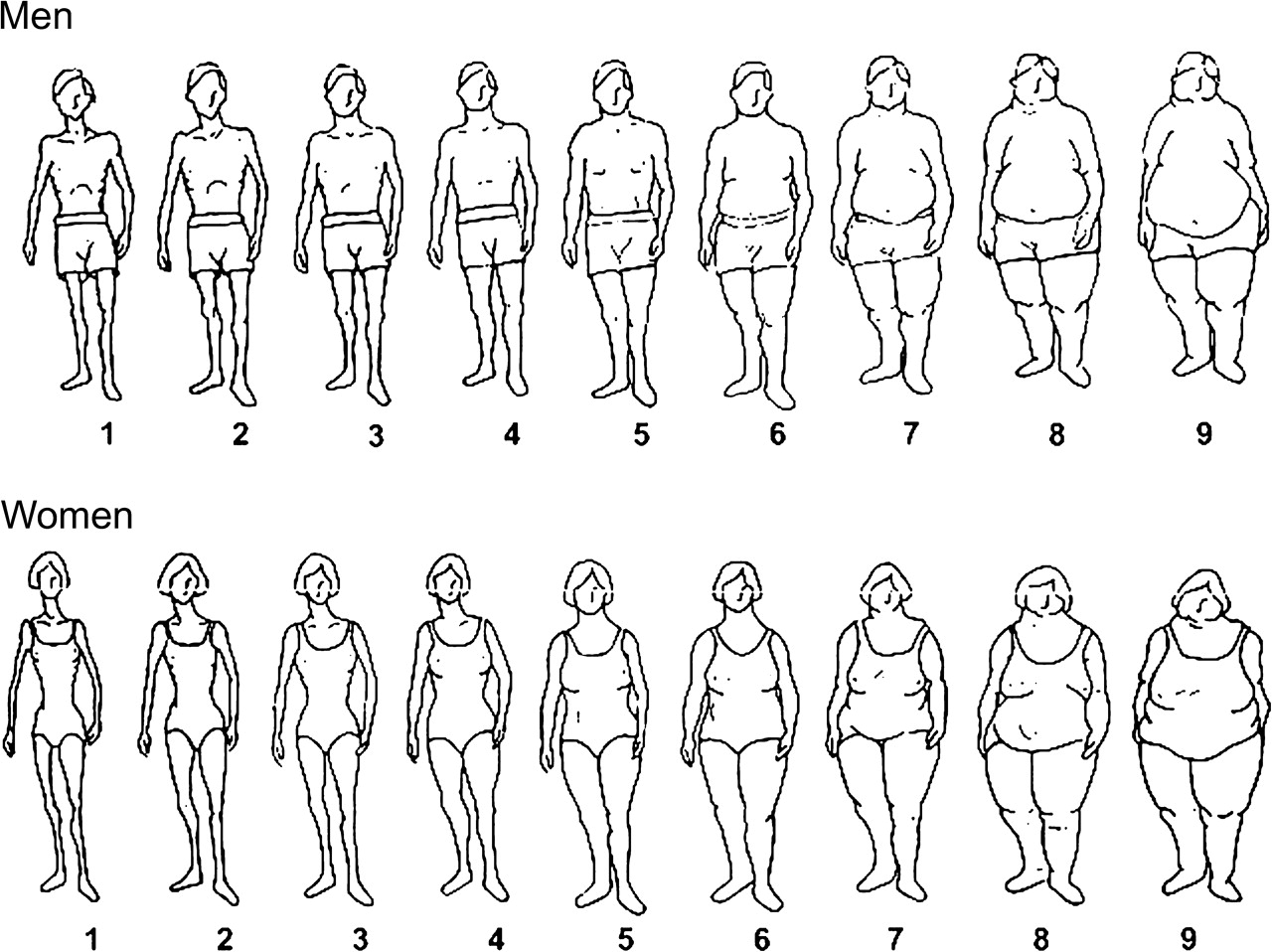


**Figure S1** Nine men or women body figures from Stunkard’s Figure Rating Scale


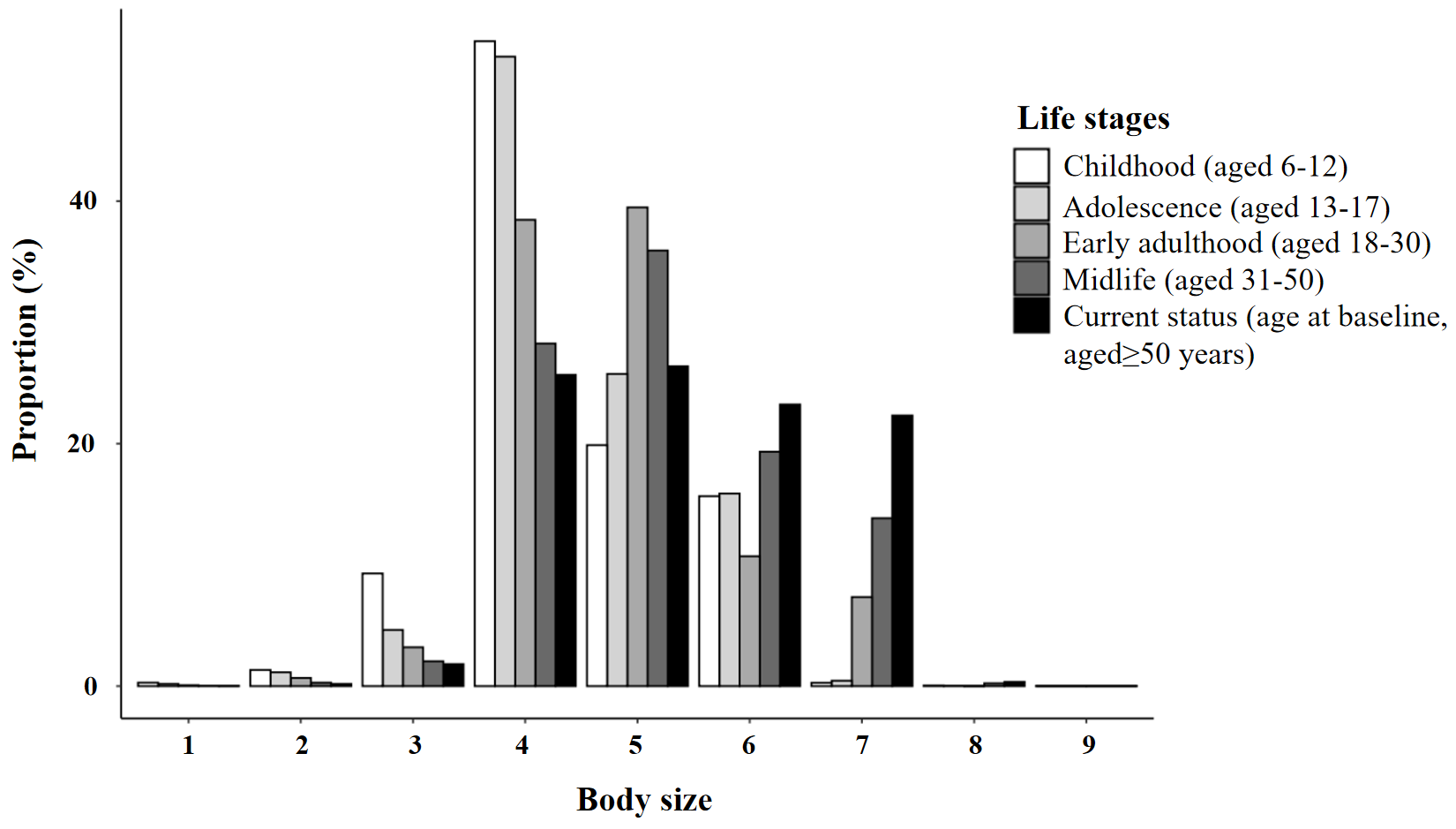


**Figure S2** Distributions of body size at different life stages


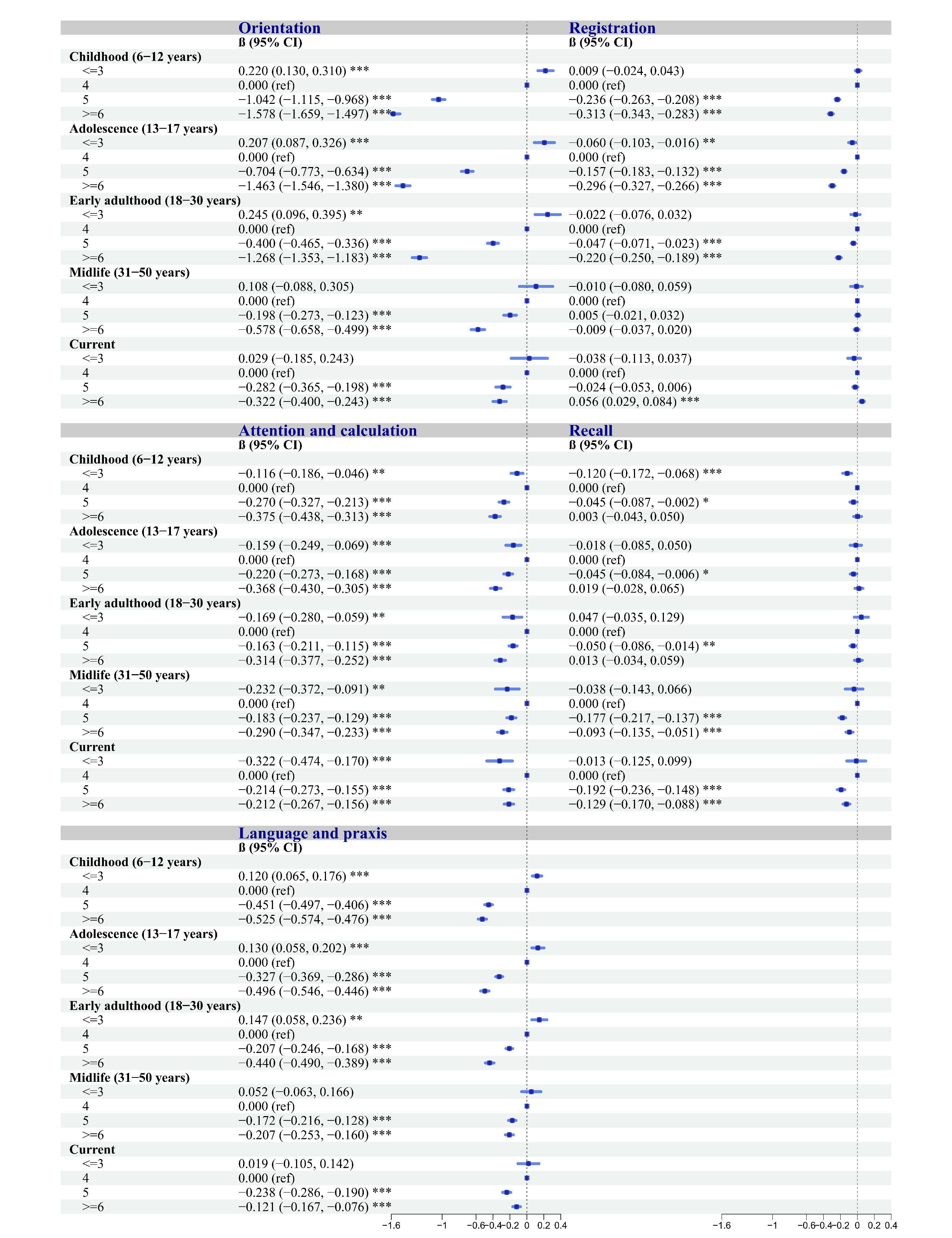


**Figure S3** Association between body size (as categorical variable) at each life stage with domain-specific cognitive function (summary of model 2)

*β*s (95% CIs) were adjusted for sex, age, education, occupation, family annual income and childhood socio-economic disadvantage.

^*^*P*<0.05, ^**^*P*<0.01, ^***^*P*<0.001

CI: confidence interval


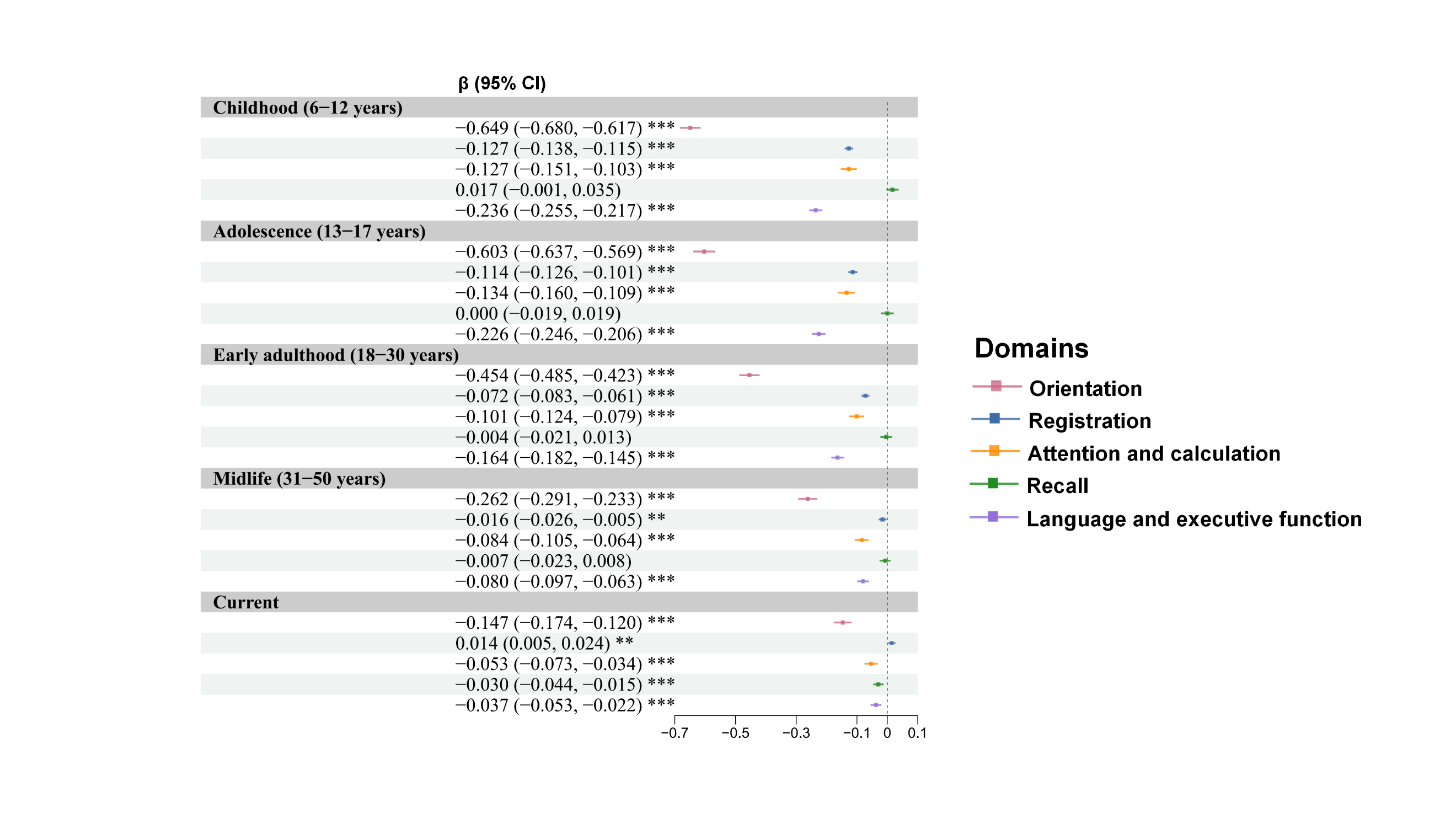


**Figure S4** Association between body size (as continuous variable) at each life stage with domain-specific cognitive function (summary of model 2)

*β*s (95% CIs) were adjusted for sex, age, education, occupation, family annual income and childhood socio-economic disadvantage.

^**^*P*<0.01, ^***^*P*<0.001

CI: confidence interval

**
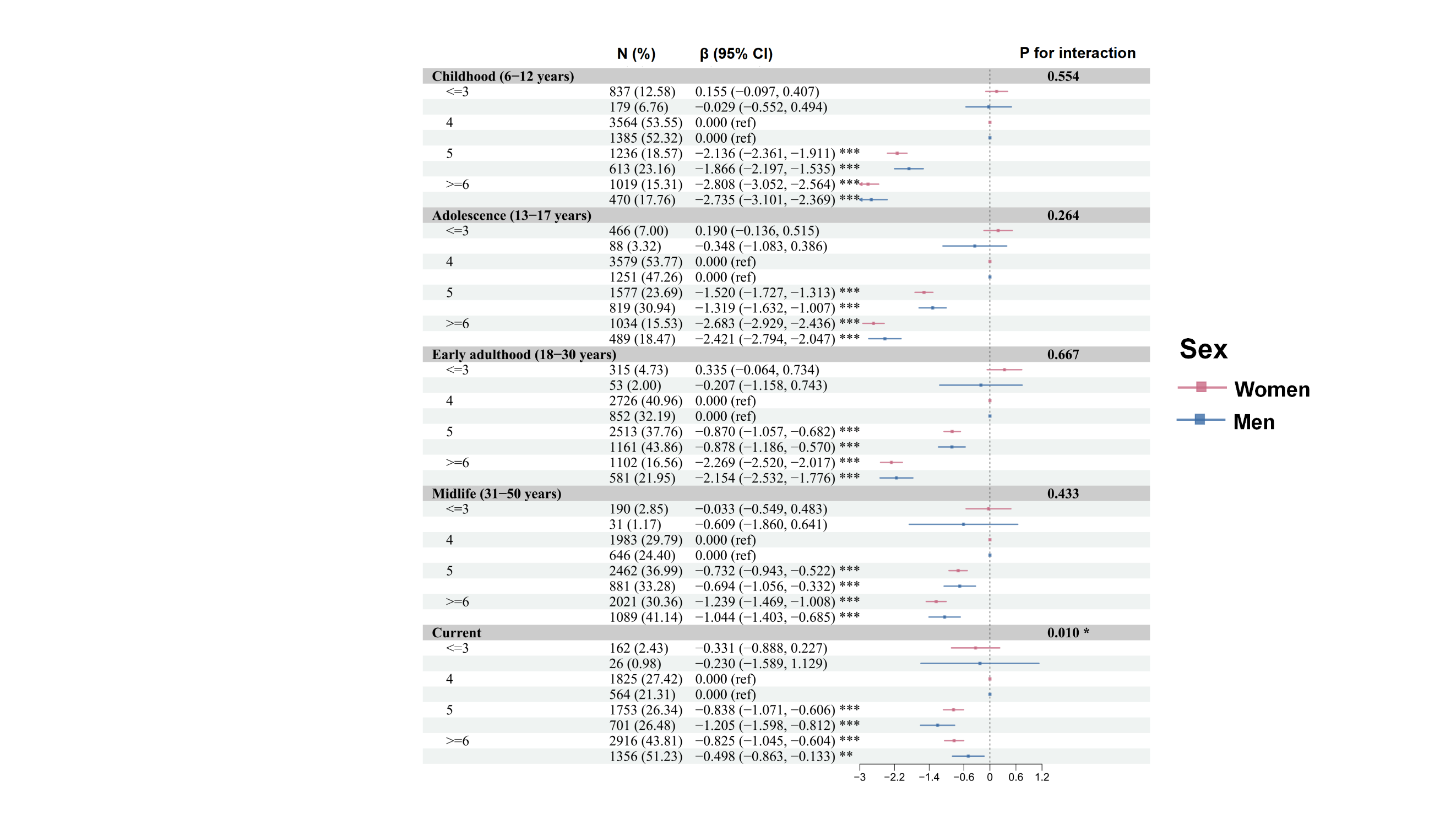
**

**Figure S5** Sex-specific associations between body size (as categorical variable) at each life stage with cognitive function

*β*s (95% CIs) were adjusted for age, education, occupation, family annual income, and childhood socio-economic disadvantage.

^**^*P*<0.01, ^***^*P*<0.001

CI: confidence interval

**
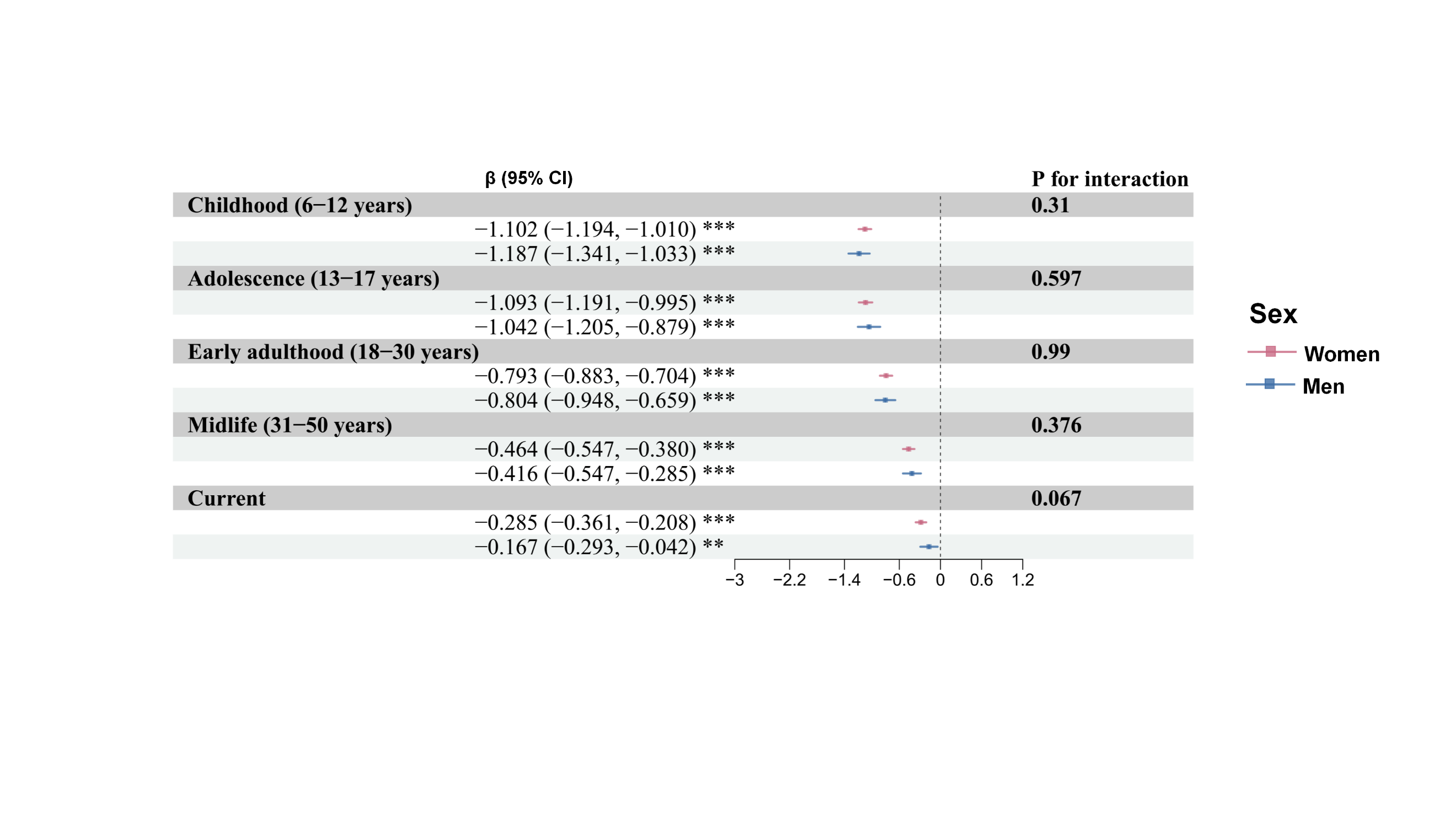
**

**Figure S6** Sex-specific associations between body size (as continuous variable) at each life stage with cognitive function

*β*s (95% CIs) were adjusted for age, education, occupation, family annual income, and childhood socio-economic disadvantage.

^**^*P*<0.01, ^***^*P*<0.001

CI: confidence interval

**
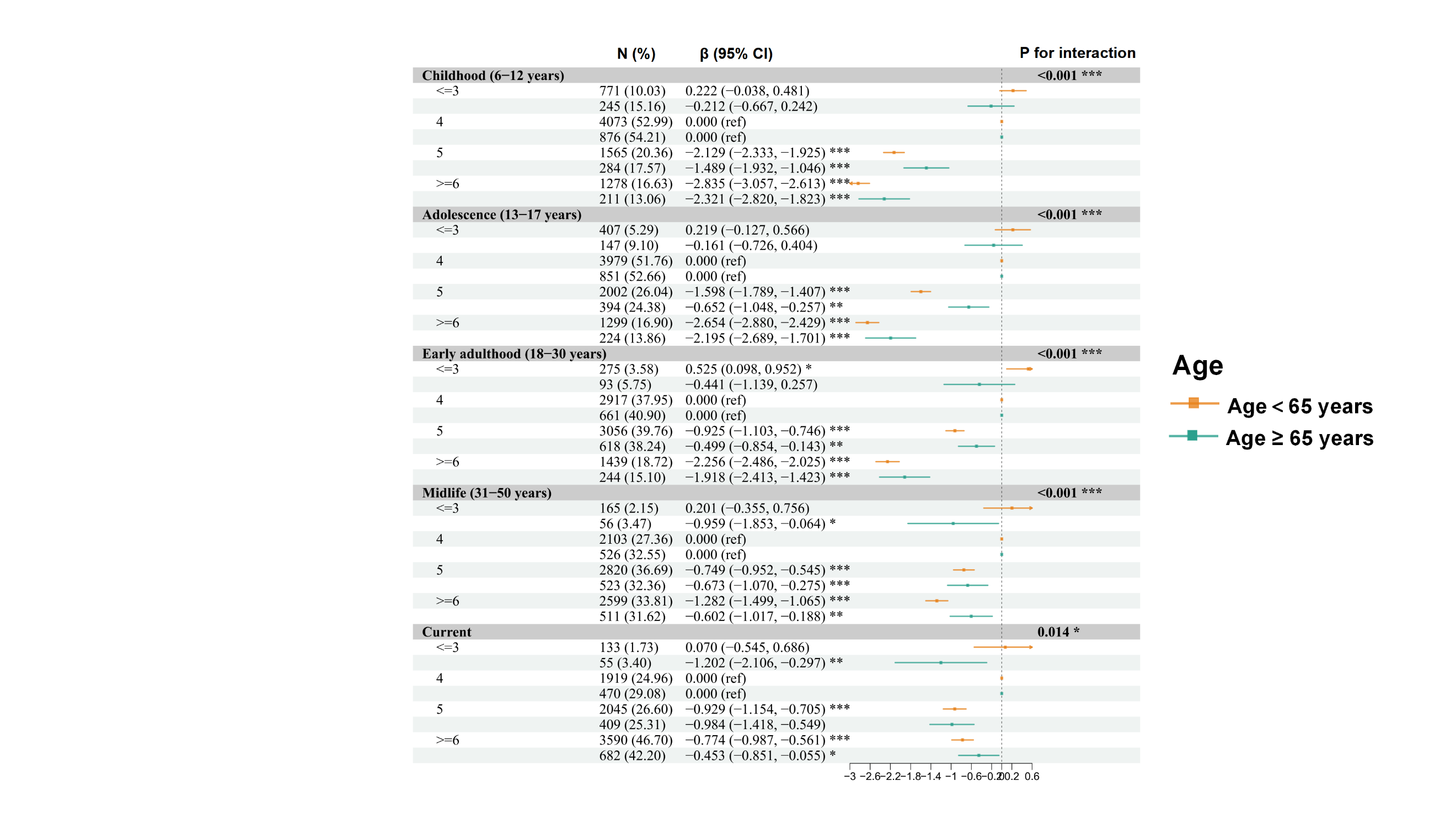
**

**Figure S7** Age-specific associations between body size (as categorical variable) at each life stage with cognitive function

*β*s (95% CIs) were adjusted for sex, education, occupation, family annual income, and childhood socio-economic disadvantage.

^*^*P*<0.05, ^**^*P*<0.01, ^***^*P*<0.001

CI: confidence interval

**
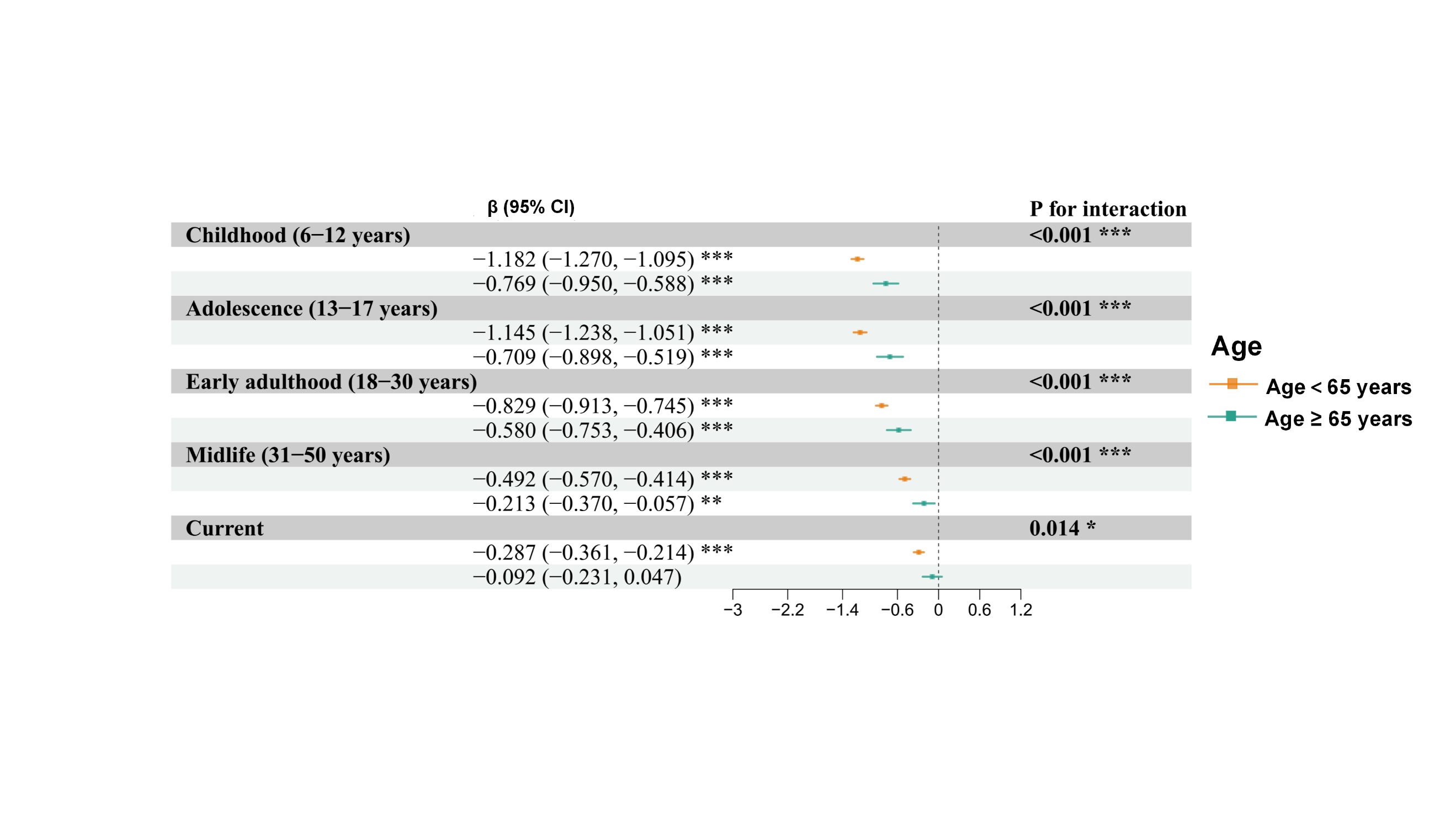
**

**Figure S8** Age-specific associations between body size (as continuous variable) at each life stage with cognitive function

*β*s (95% CIs) were adjusted for sex, education, occupation, family annual income, and childhood socio-economic disadvantage.

^*^*P*<0.05, ^**^*P*<0.01, ^***^*P*<0.001

CI: confidence interval


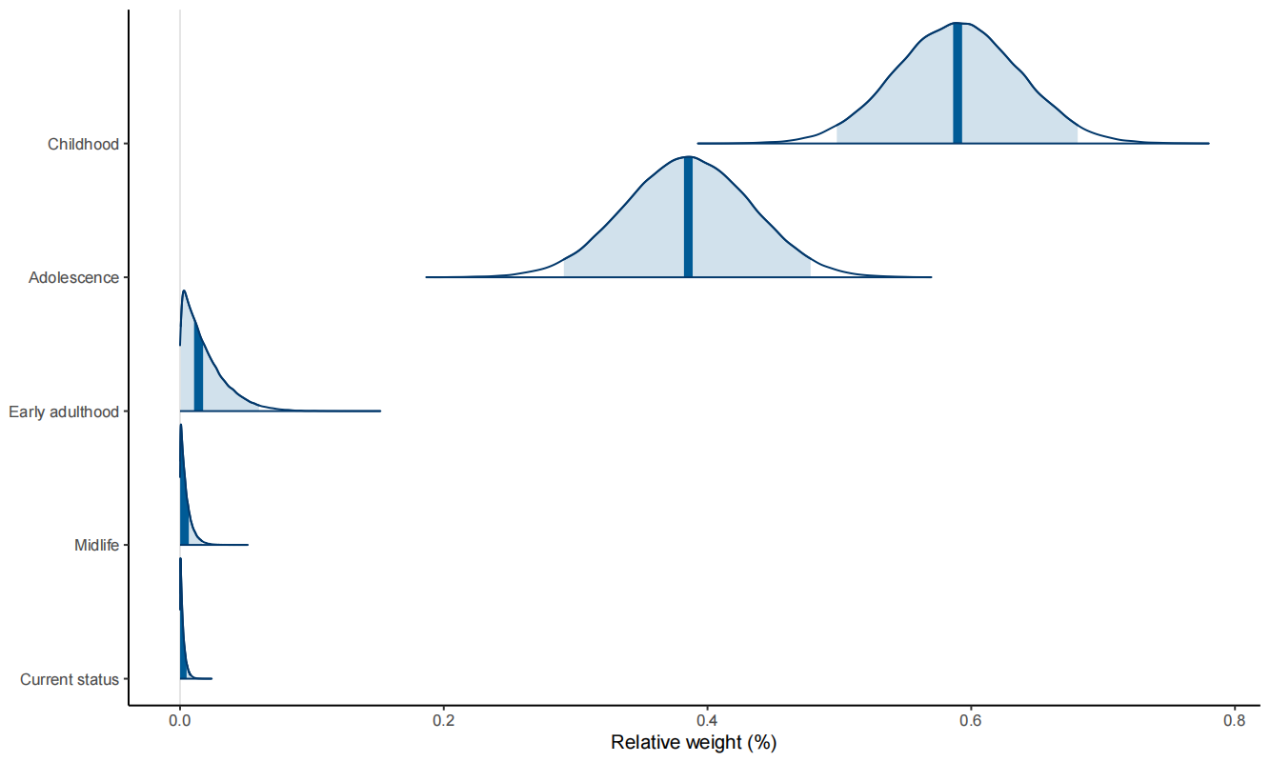


**Figure S9** Posterior distributions of relative weights across life stages on midlife and late-life cognitive function

Note: The blue vertical lines represented means of relative weight, and the shaded areas represented 95% credible interval of relative weight.


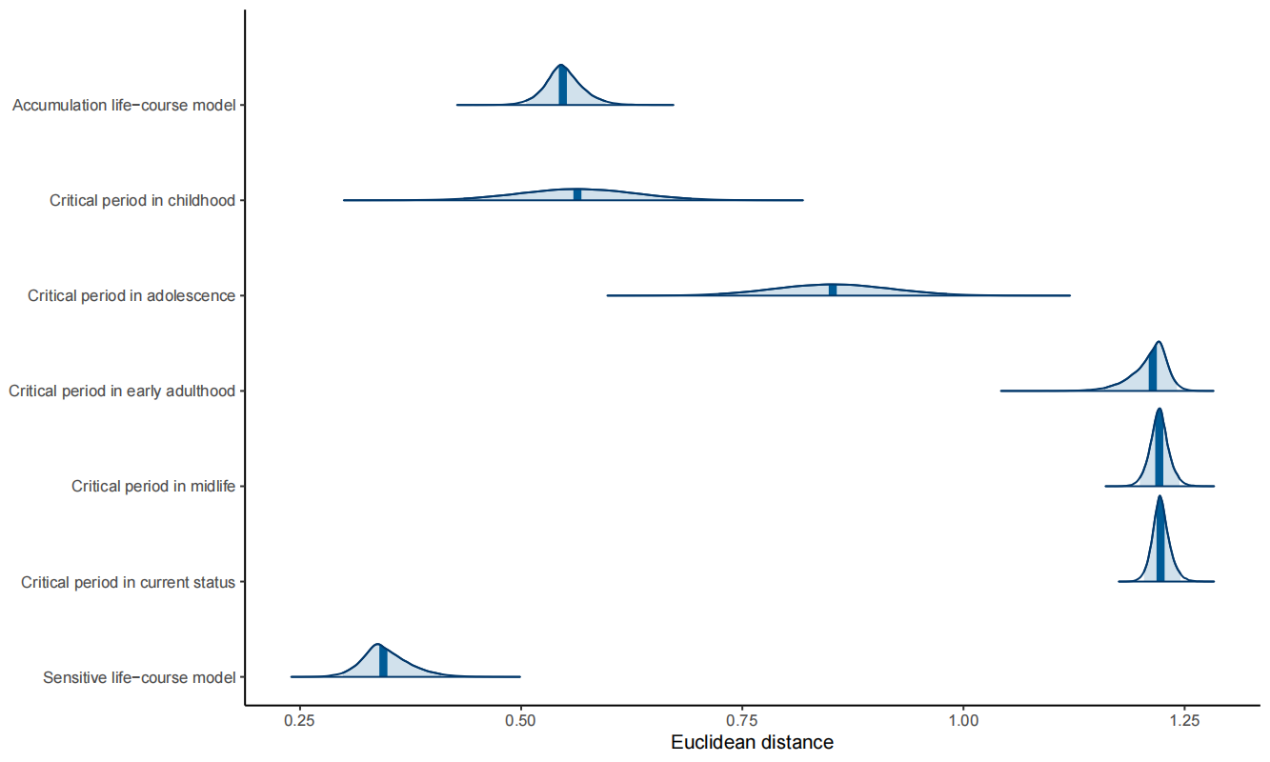


**Figure S10** Posterior distributions of Euclidean distance between the expected and estimated weights of body size on midlife and late-life cognitive function

Note: The blue vertical lines represented means of Euclidean distance, and the shaded areas represented 95% credible interval of Euclidean distance.


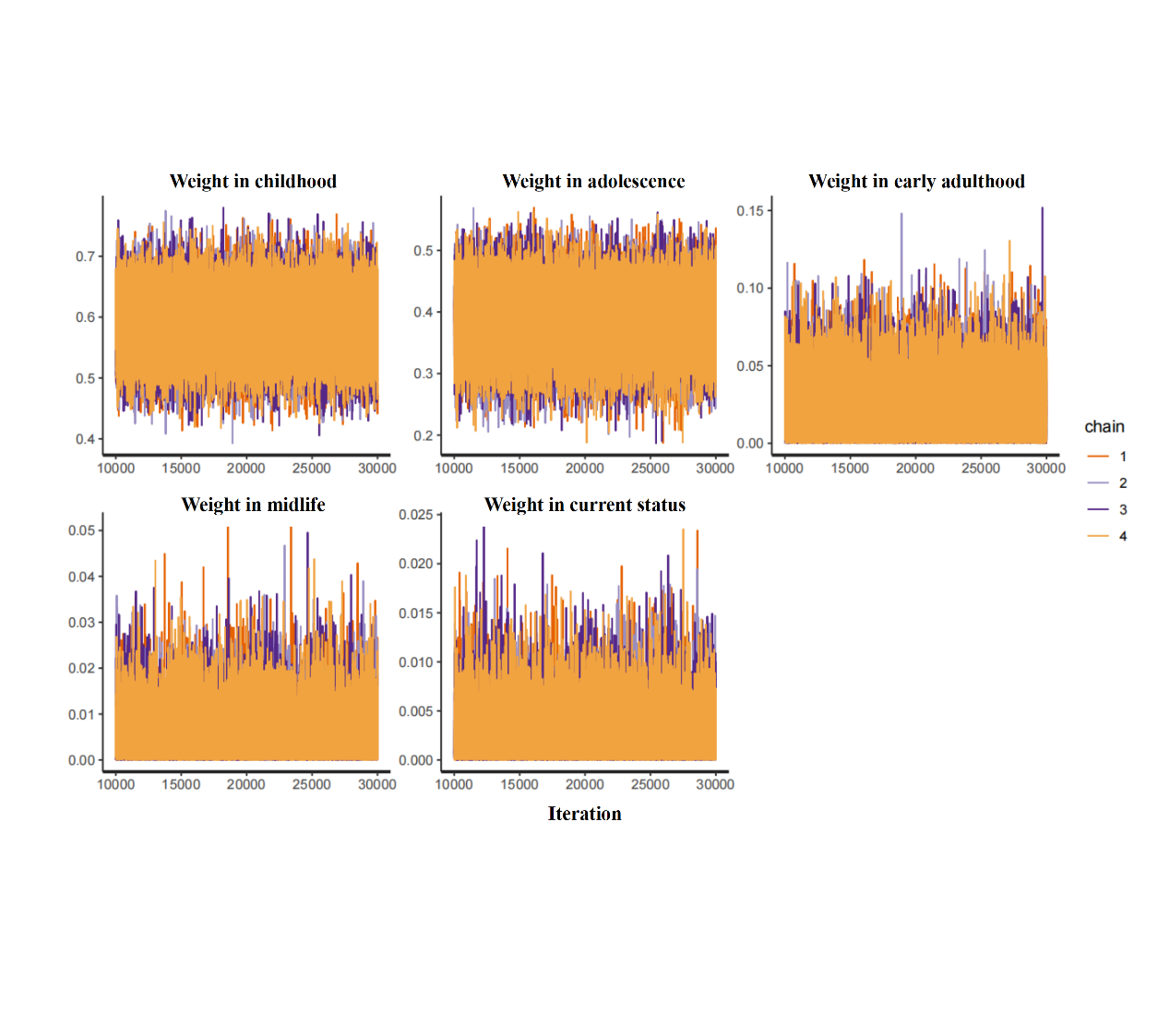


**Figure S11** Trace plots of the four parallel Hamiltonian Monte Carlo chains based on Bayesian relevant life course exposure model
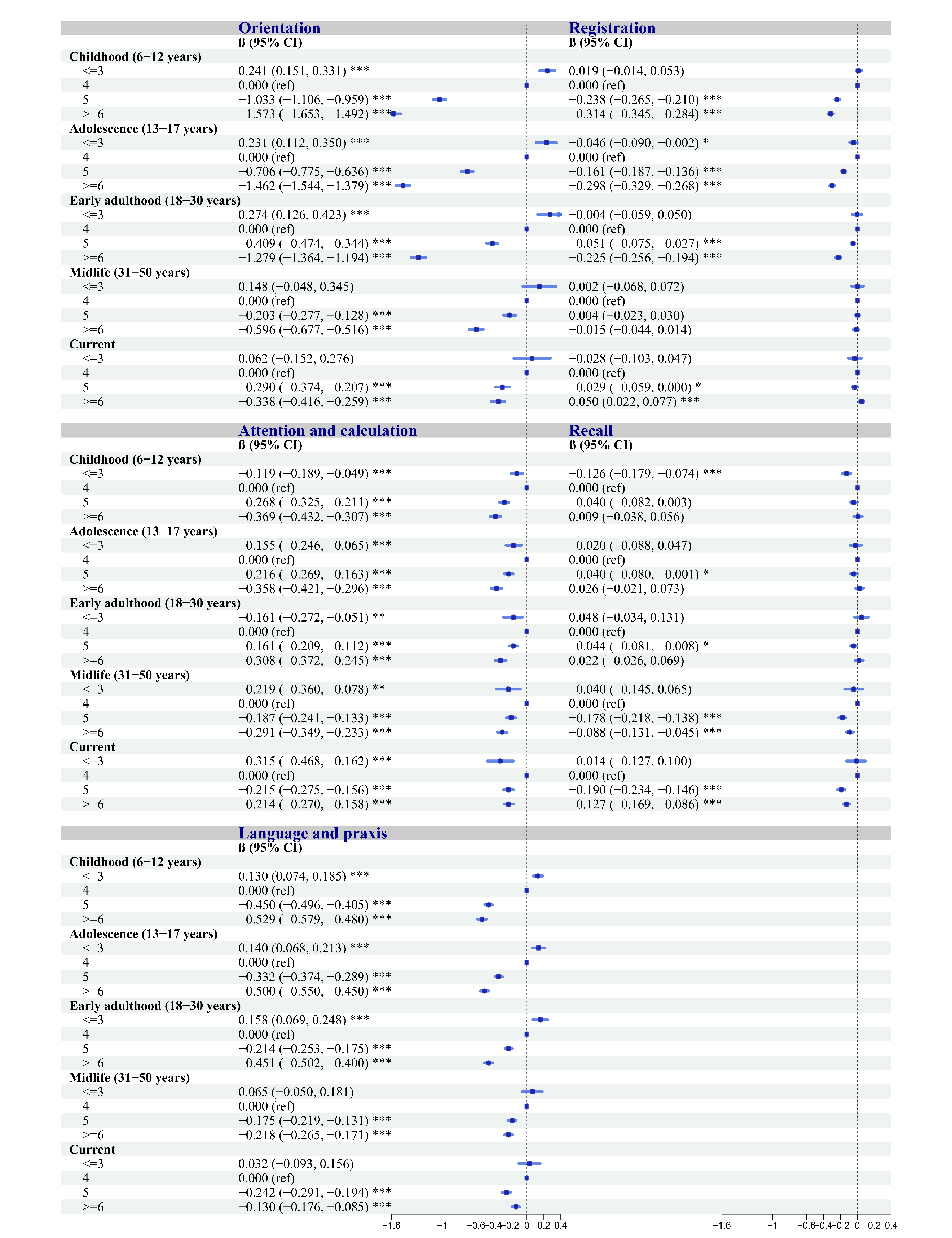


**Figure S12** Association between body size (as categorical variable) at each life stage with domain-specific cognitive function further adjusting for current body mass index (summary of model 2)

*β*s (95% CIs) were adjusted for sex, age, education, occupation, family annual income, childhood socio-economic disadvantage and current body mass index.

^*^*P*<0.05, ^**^*P*<0.01, ^***^*P*<0.001

CI: confidence interval


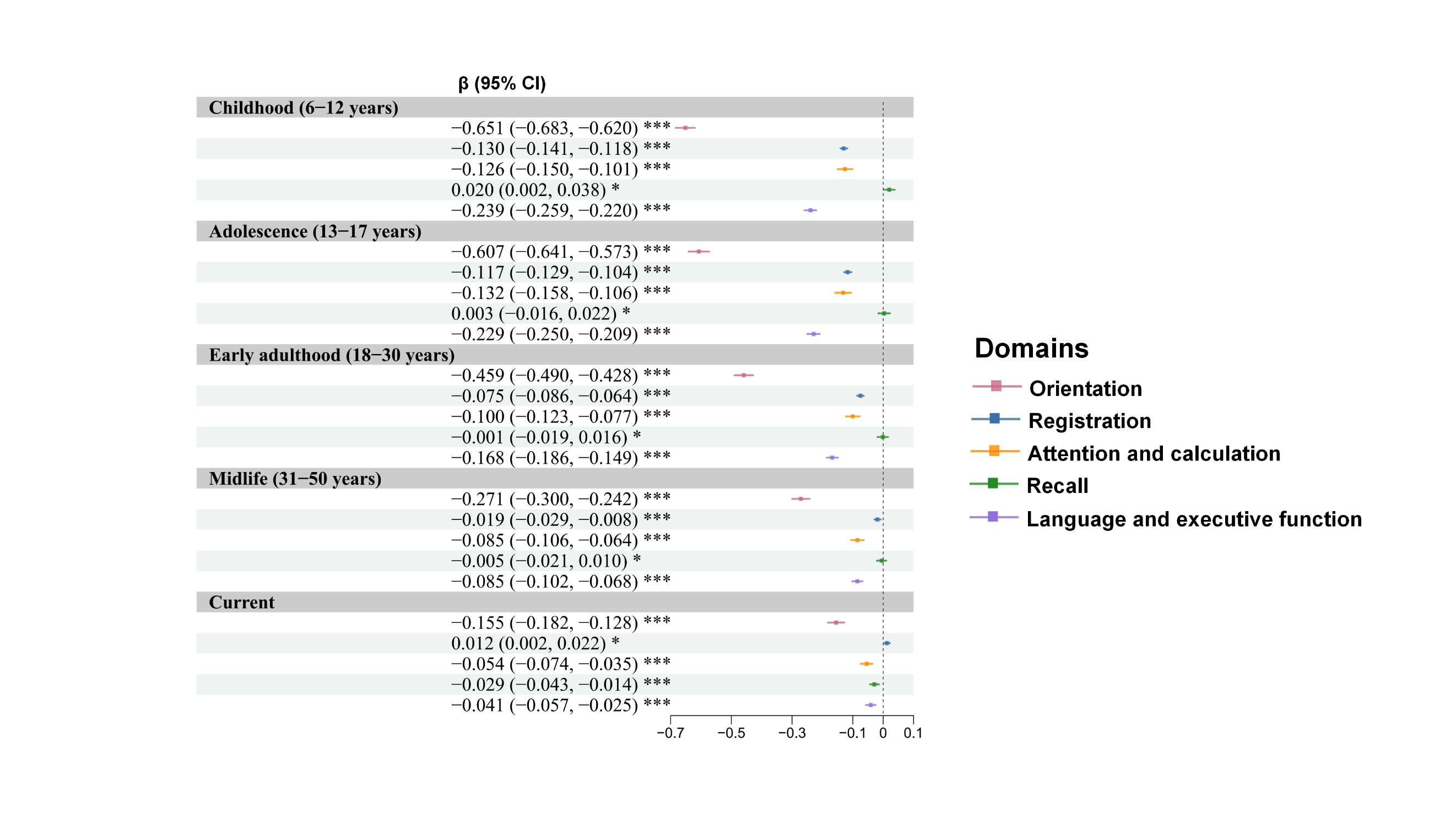


**Figure S13** Association between body size (as continuous variable) at each life stage with domain-specific cognitive function further adjusting for current body mass index (summary of model 2)

*β*s (95% CIs) were adjusted for sex, age, education, occupation, family annual income, childhood socio-economic disadvantage and current body mass index.

^**^*P*<0.01, ^***^*P*<0.001

CI: confidence interval**
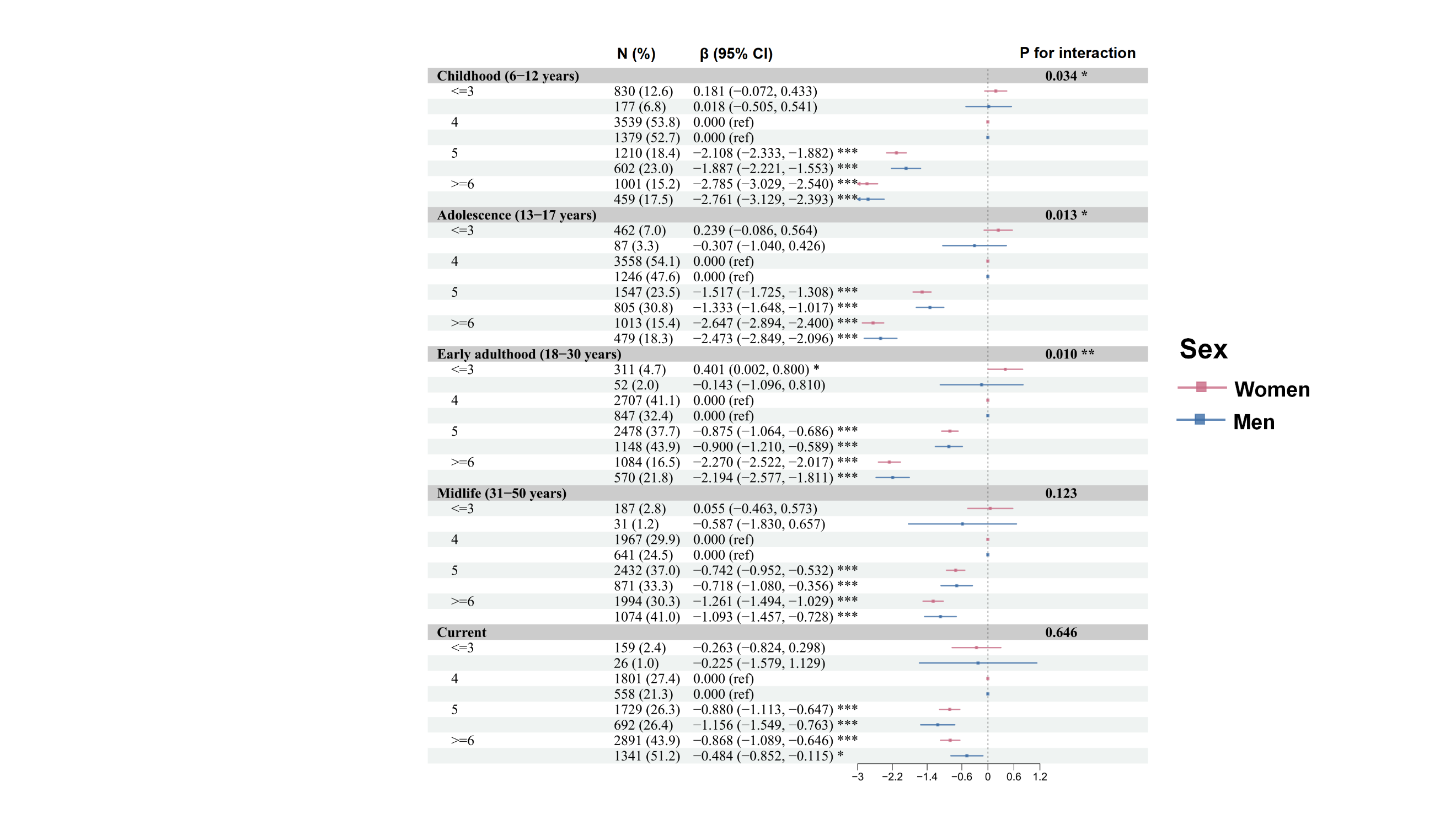
**

**Figure S14** Sex-specific associations between body size (as categorical variable) at each life stage with cognitive function further adjusting for current body mass index

*β*s (95% CIs) were adjusted for age, education, occupation, family annual income, childhood socio-economic disadvantage and current body mass index.

^*^*P*<0.05, ^**^*P*<0.01, ^***^*P*<0.001

CI: confidence interval

**
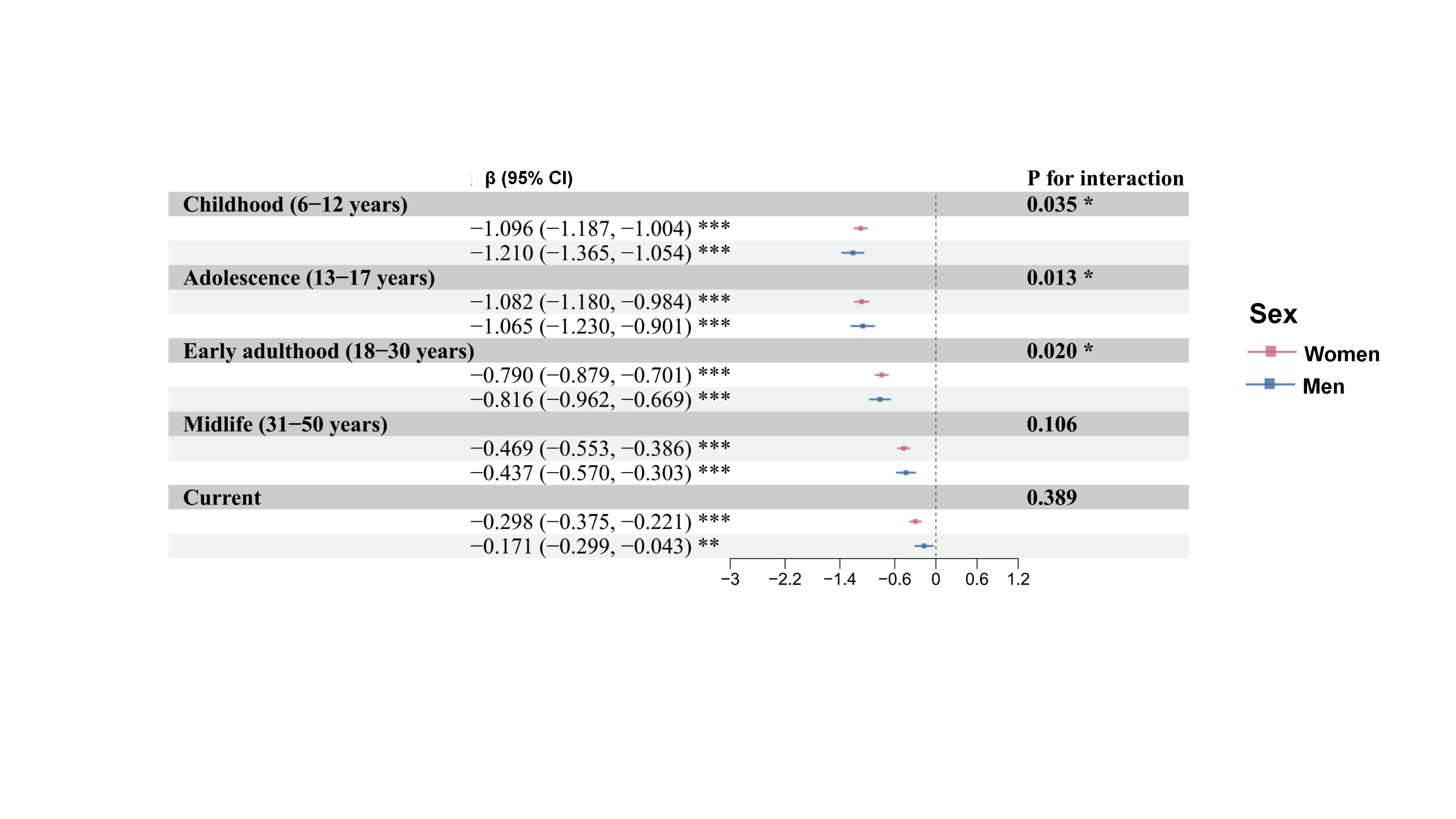
**

**Figure S15** Sex-specific associations between body size (as continuous variable) at each life stage with cognitive function further adjusting for current body mass index

*β*s (95% CIs) were adjusted for age, education, occupation, family annual income, childhood socio-economic disadvantage and current body mass index.

^*^*P*<0.05, ^**^*P*<0.01, ^***^*P*<0.001

CI: confidence interval


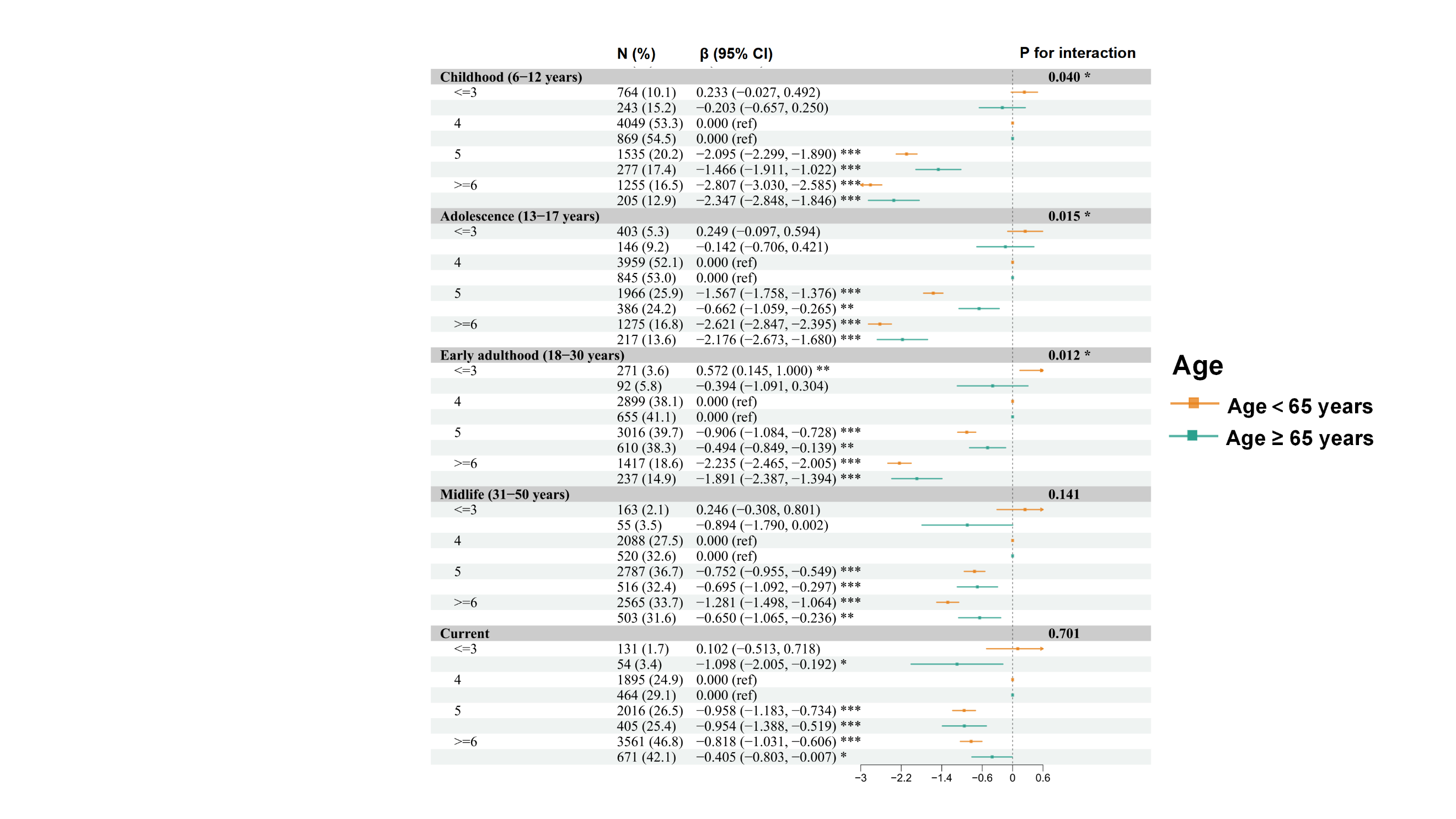


**Figure S16** Age-specific associations between body size (as categorical variable) at each life stage with cognitive function further adjusting for current body mass index

*β*s (95% CIs) were adjusted for sex, education, occupation, family annual income, childhood socio-economic disadvantage and current body mass index.

^*^*P*<0.05, ^**^*P*<0.01, ^***^*P*<0.001

CI: confidence interval


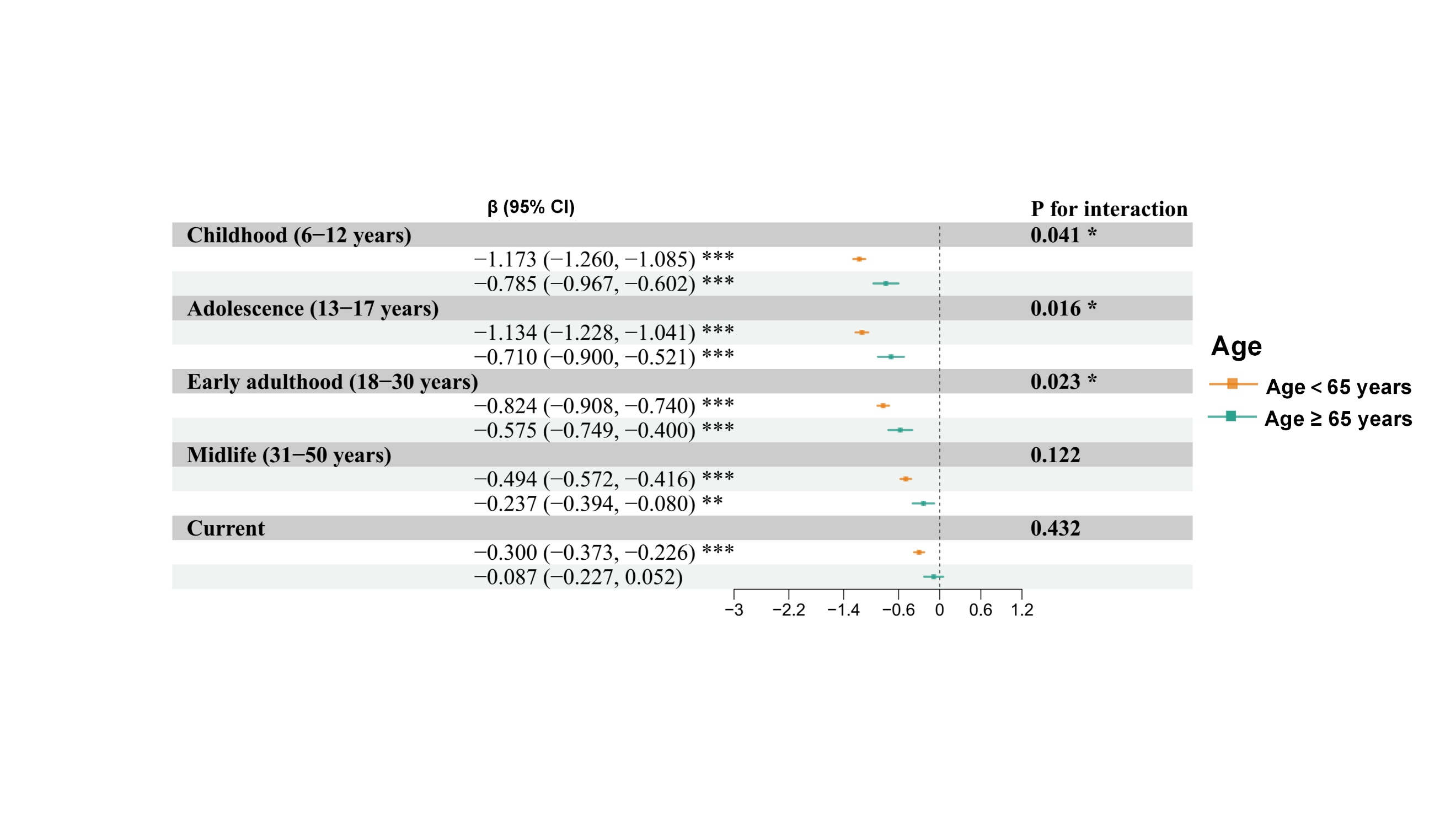


**Figure S17** Age-specific associations between body size (as continuous variable) at each life stage with cognitive function further adjusting for current body mass index

*β*s (95% CIs) were adjusted for sex, education, occupation, family annual income, childhood socio-economic disadvantage and current body mass index.

^*^*P*<0.05, ^**^*P*<0.01, ^***^*P*<0.001

CI: confidence interval


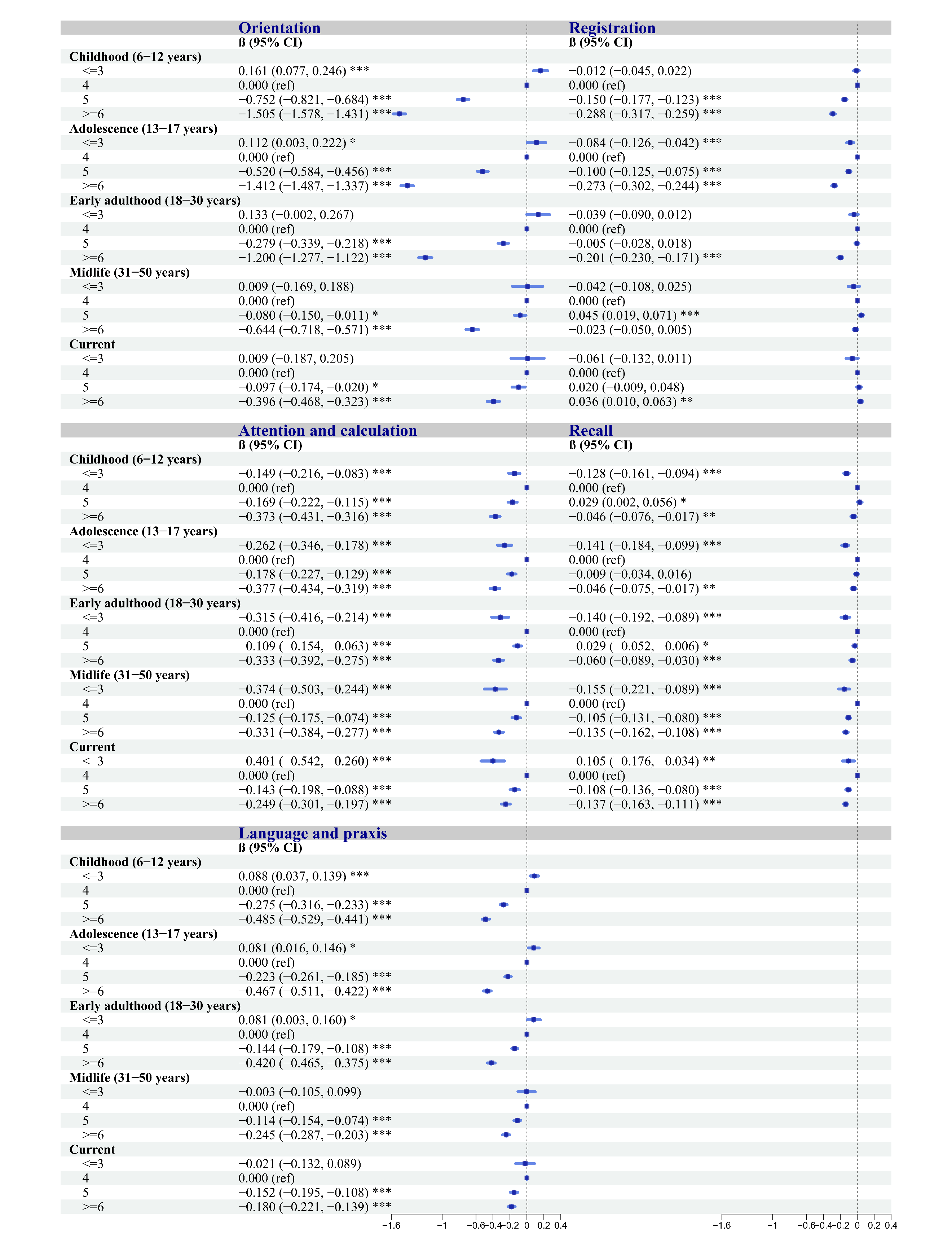


**Figure S18** Association between body size (as categorical variable) at each life stage with domain-specific cognitive function after excluding those with an MMSE recall-domain score < 2 (summary of model 2)

*β*s (95% CIs) were adjusted for sex, age, education, occupation, family annual income and childhood socio-economic disadvantage.

^*^*P*<0.05, ^**^*P*<0.01, ^***^*P*<0.001

CI: confidence interval


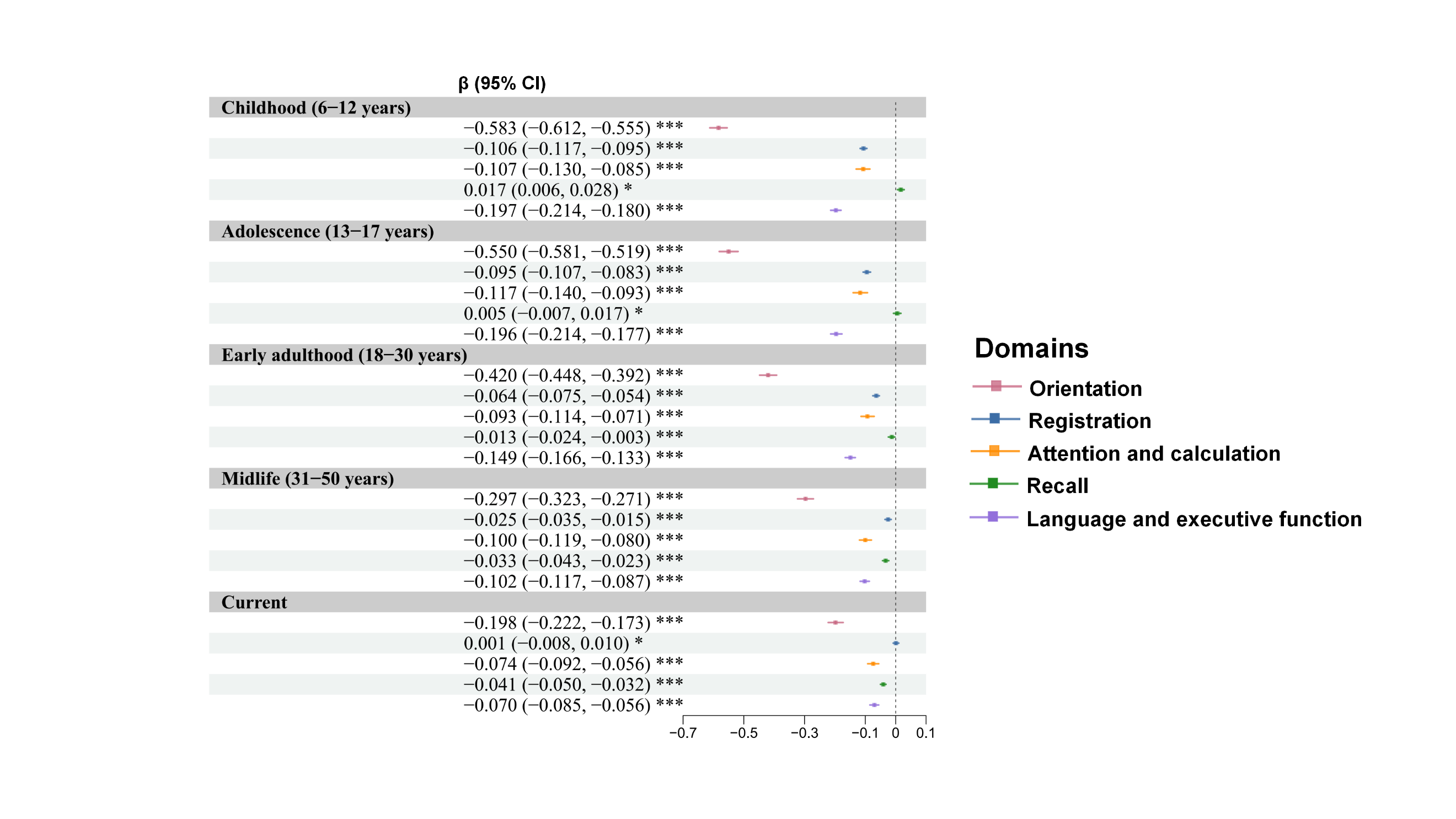


**Figure S19** Association between body size (as continuous variable) at each life stage with domain-specific cognitive function after excluding those with an MMSE recall-domain score < 2 (summary of model 2)

*β*s (95% CIs) were adjusted for sex, age, education, occupation, family annual income and childhood socio-economic disadvantage.

^*^*P*<0.05, ^***^*P*<0.001

CI: confidence interval
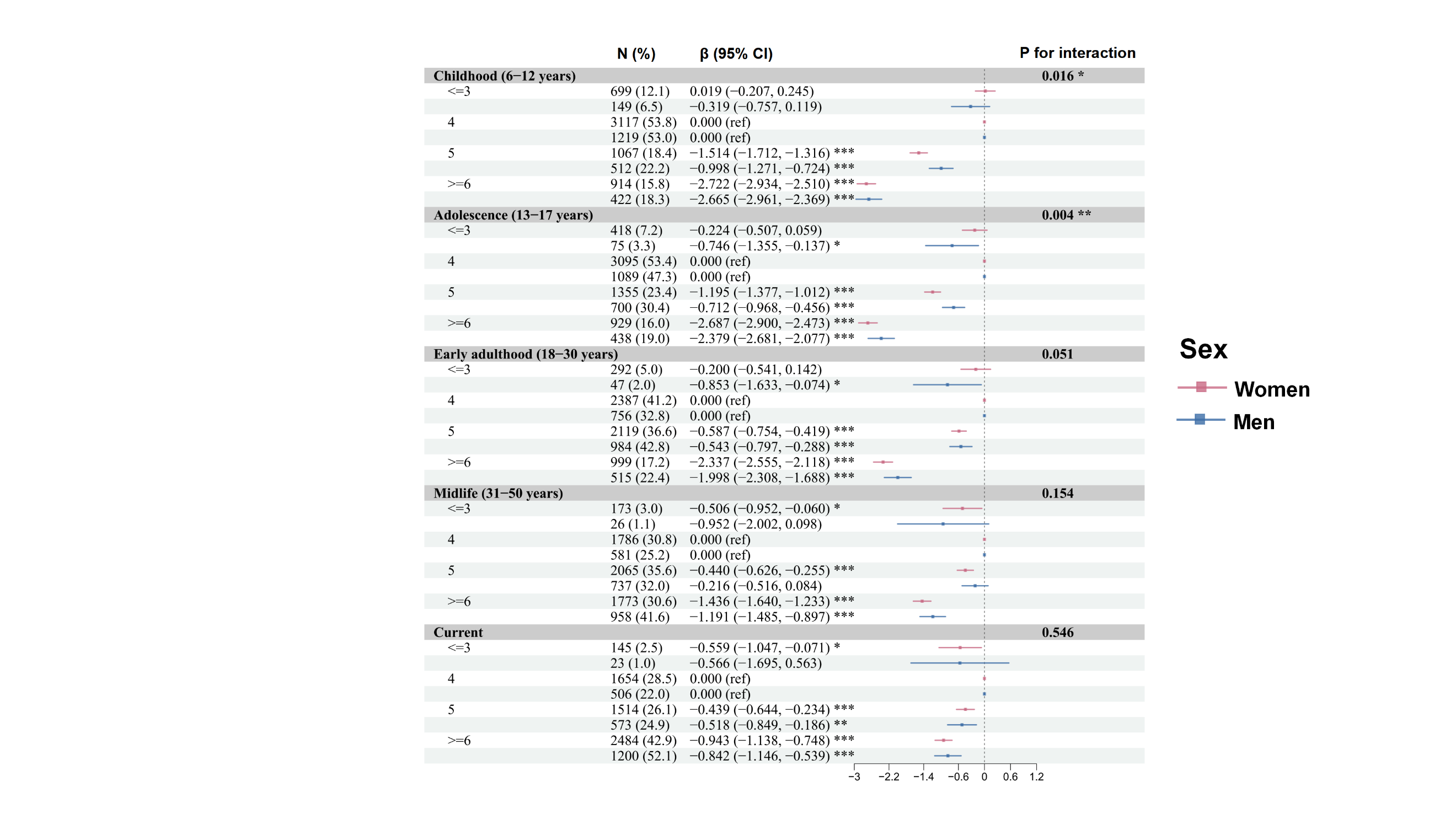


**Figure S20** Sex-specific associations between body size (as categorical variable) at each life stage with cognitive function after excluding those with an MMSE recall-domain score < 2

*β*s (95% CIs) were adjusted for age, education, occupation, family annual income and childhood socio-economic disadvantage.

^*^*P*<0.05, ^**^*P*<0.01, ^***^*P*<0.001

CI: confidence interval


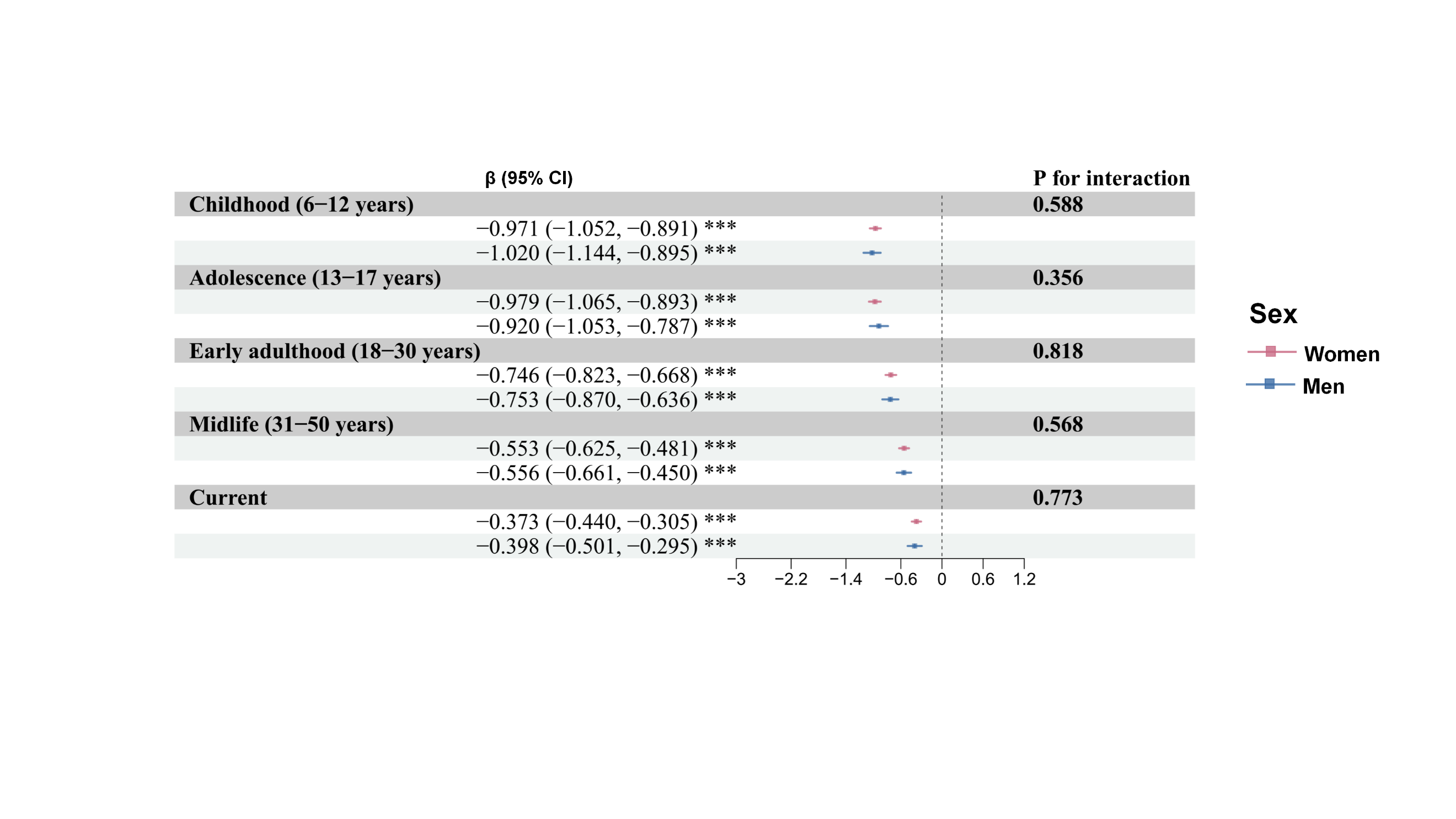


**Figure S21** Sex-specific associations between body size (as continuous variable) at each life stage with cognitive function after excluding those with an MMSE recall-domain score < 2

*β*s (95% CIs) were adjusted for age, education, occupation, family annual income and childhood socio-economic disadvantage.

^***^*P*<0.001


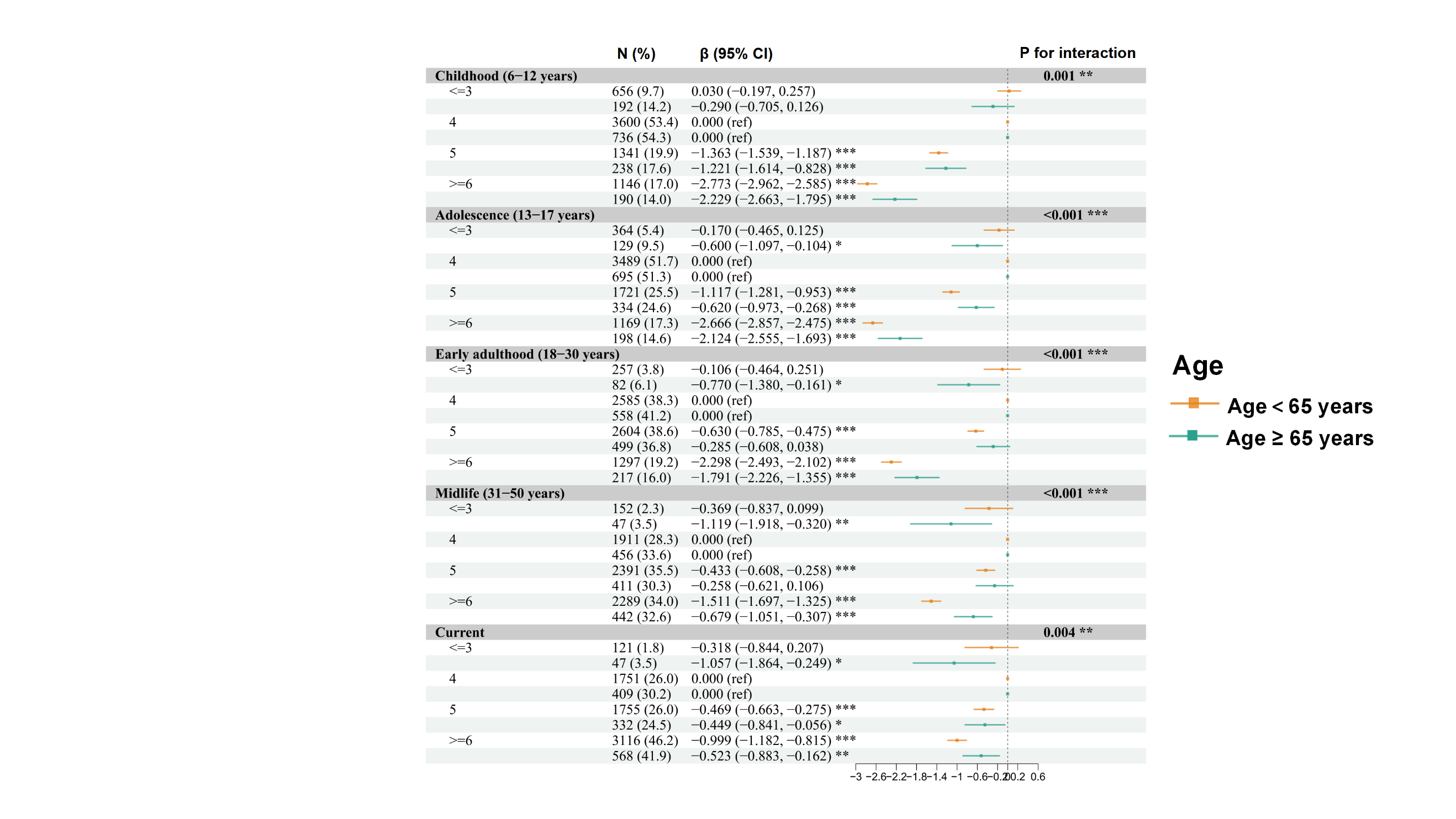


**Figure S22** Age-specific associations between body size (as categorical variable) at each life stage with cognitive function after excluding those with an MMSE recall-domain score < 2

*β*s (95% CIs) were adjusted for sex, education, occupation, family annual income and childhood socio-economic disadvantage.

^*^*P*<0.05, ^**^*P*<0.01, ^***^*P*<0.001

CI: confidence interval


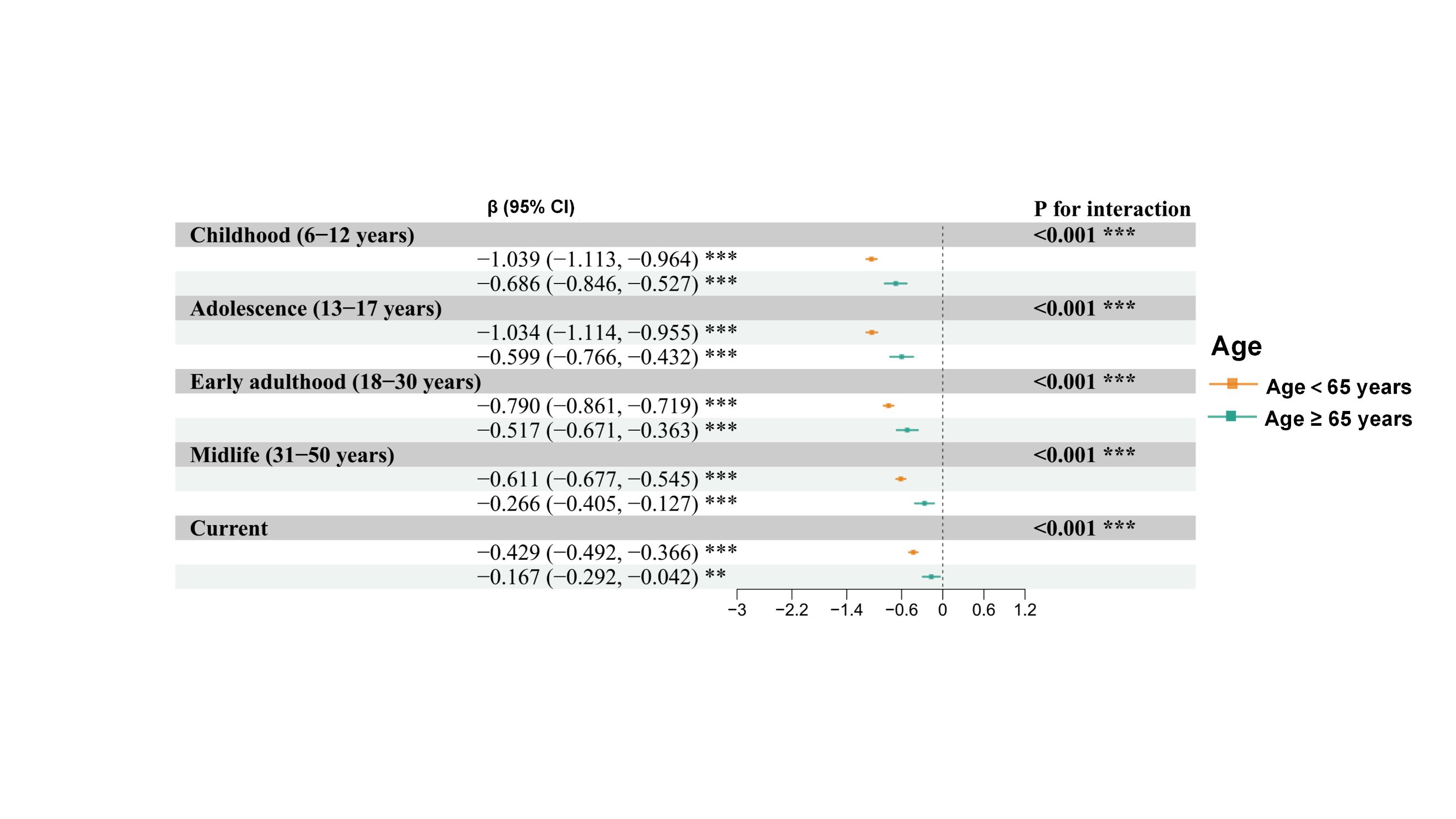


**Figure S23** Age-specific associations between body size (as continuous variable) at each life stage with cognitive function after excluding those with an MMSE recall-domain score < 2

*β*s (95% CIs) were adjusted for sex, education, occupation, family annual income and childhood socio-economic disadvantage.

^**^*P*<0.01, ^***^*P*<0.001

CI: confidence interval

**Table S1** Association between body size at each life stage with the orientation domain

|  | N (%) | Crude model,  β (95% CI) | Model 1,  β (95% CI) † | Model 2,  β (95% CI) ‡ |
| --- | --- | --- | --- | --- |
| Childhood (6-12 years) | | | | |
| ≤3 (smallest figures) | 1016 (10.92) | 0.091 (-0.006, 0.188) | 0.097 (0.000, 0.195) | 0.220 (0.130, 0.310) ^***^ |
| 4 | 4949 (53.20) | 0.000 (ref) | 0.000 (ref) | 0.000 (ref) |
| 5 | 1849 (19.88) | -1.241 (-1.318, -1.164) ^***^ | -1.245 (-1.321, -1.168) ^***^ | -1.042 (-1.115, -0.968) ^***^ |
| ≥6 (largest figures) | 1489 (16.01) | -1.949 (-2.032, -1.866) | -1.949 (-2.032, -1.866) ^***^ | -1.578 (-1.659, -1.497) ^***^ |
| *P*_trend_ |  | <0.001 | <0.001 | <0.001 |
| Per 1-figure increase |  | -0.770 (-0.802, -0.738) ^***^ | -0.773 (-0.805, -0.741) ^***^ | -0.649 (-0.680, -0.617) ^***^ |
| Adolescence (13-17 years) | | | | |
| ≤3 (smallest figures) | 554 (5.96) | 0.043 (-0.087, 0.172) | 0.043 (-0.087, 0.173) | 0.207 (0.087, 0.326) ^***^ |
| 4 | 4830 (51.92) | 0.000 (ref) | 0.000 (ref) | 0.000 (ref) |
| 5 | 2396 (25.76) | -0.963 (-1.035, -0.891) ^***^ | -0.973 (-1.045, -0.901) ^***^ | -0.704 (-0.773, -0.634) ^***^ |
| ≥6 (largest figures) | 1523 (16.37) | -1.885 (-1.970, -1.800) ^***^ | -1.888 (-1.973, -1.803) ^***^ | -1.463 (-1.546, -1.380) ^***^ |
| *P*_trend_ |  | <0.001 | <0.001 | <0.001 |
| Per 1-figure increase |  | -0.762 (-0.796, -0.728) ^***^ | -0.766 (-0.801, -0.732) ^***^ | -0.603 (-0.637, -0.569) ^***^ |
| Early adulthood (18-30 years) | | | | |
| ≤3 (smallest figures) | 368 (3.96) | 0.084 (-0.079, 0.247) | 0.086 (-0.077, 0.249) | 0.245 (0.096, 0.395) ^**^ |
| 4 | 3578 (38.46) | 0.000 (ref) | 0.000 (ref) | 0.000 (ref) |
| 5 | 3674 (39.49) | -0.539 (-0.609, -0.469) ^***^ | -0.548 (-0.618, -0.478) ^***^ | -0.400 (-0.465, -0.336) ^***^ |
| ≥6 (largest figures) | 1683 (18.09) | -1.711 (-1.799, -1.623) ^***^ | -1.719 (-1.807, -1.631) ^***^ | -1.268 (-1.353, -1.183) ^***^ |
| *P*_trend_ |  | <0.001 | <0.001 | <0.001 |
| Per 1-figure increase |  | -0.581 (-0.613, -0.548) ^***^ | -0.584 (-0.616, -0.551) ^***^ | -0.454 (-0.485, -0.423) ^***^ |
| Midlife (31-50 years) | | | | |
| ≤3 (smallest figures) | 221 (2.38) | 0.055 (-0.163, 0.274) | 0.052 (-0.167, 0.271) | 0.108 (-0.088, 0.305) |
| 4 | 2629 (28.26) | 0.000 (ref) | 0.000 (ref) | 0.000 (ref) |
| 5 | 3343 (35.93) | -0.434 (-0.515, -0.353) ^***^ | -0.426 (-0.507, -0.344) ^***^ | -0.198 (-0.273, -0.123) ^***^ |
| ≥6 (largest figures) | 3110 (33.43) | -0.932 (-1.014, -0.849) ^***^ | -0.933 (-1.016, -0.850) ^***^ | -0.578 (-0.658, -0.499) ^***^ |
| *P*_trend_ |  | <0.001 | <0.001 | <0.001 |
| Per 1-figure increase |  | -0.397 (-0.427, -0.367) ^***^ | -0.397 (-0.428, -0.367) ^***^ | -0.262 (-0.291, -0.233) ^***^ |
| Current | | | | |
| ≤3 (smallest figures) | 188 (2.02) | -0.012 (-0.252, 0.227) | -0.031 (-0.270, 0.209) | 0.029 (-0.185, 0.243) |
| 4 | 2389 (25.68) | 0.000 (ref) | 0.000 (ref) | 0.000 (ref) |
| 5 | 2454 (26.38) | -0.730 (-0.821, -0.640) ^***^ | -0.724 (-0.815, -0.633) ^***^ | -0.282 (-0.365, -0.198) ^***^ |
| ≥6 (largest figures) | 4272 (45.92) | -0.572 (-0.653, -0.491) ^***^ | -0.567 (-0.648, -0.486) ^***^ | -0.322 (-0.400, -0.243) ^***^ |
| *P*_trend_ |  | <0.001 | <0.001 | <0.001 |
| Per 1-figure increase |  | -0.195 (-0.224, -0.167) ^***^ | -0.193 (-0.221, -0.165) ^***^ | -0.147 (-0.174, -0.120) ^***^ |

CI: confidence interval

^†^ Model 1 were adjusted for sex, age.

‡ Model 2 were additionally adjusted for education, occupation, family annual income and childhood socio-economic disadvantage.

^**^*P*<0.01, ^***^*P*<0.001

**Table S2** Association between body size at each life stage with the registration domain

|  | N (%) | Crude model,  β (95% CI) | Model 1,  β (95% CI) † | Model 2,  β (95% CI) ‡ |
| --- | --- | --- | --- | --- |
| Childhood (6-12 years) | | | | |
| ≤3 (smallest figures) | 1016 (10.92) | -0.019 (-0.053, 0.016) | -0.018 (-0.053, 0.016) | 0.009 (-0.024, 0.043) |
| 4 | 4949 (53.20) | 0.000 (ref) | 0.000 (ref) | 0.000 (ref) |
| 5 | 1849 (19.88) | -0.252 (-0.280, -0.225) ^***^ | -0.253 (-0.280, -0.225) ^***^ | -0.236 (-0.263, -0.208) ^***^ |
| ≥6 (largest figures) | 1489 (16.01) | -0.355 (-0.385, -0.326) ^***^ | -0.356 (-0.385, -0.326) ^***^ | -0.313 (-0.343, -0.283) ^***^ |
| *P*_trend_ |  | <0.001 | <0.001 | <0.001 |
| Per 1-figure increase |  | -0.139 (-0.150, -0.128) ^***^ | -0.139 (-0.151, -0.128) ^***^ | -0.127 (-0.138, -0.115) ^***^ |
| Adolescence (13-17 years) | | | | |
| ≤3 (smallest figures) | 554 (5.96) | -0.097 (-0.142, -0.052) ^***^ | -0.098 (-0.143, -0.052) ^***^ | -0.060 (-0.103, -0.016) ^**^ |
| 4 | 4830 (51.92) | 0.000 (ref) | 0.000 (ref) | 0.000 (ref) |
| 5 | 2396 (25.76) | -0.191 (-0.216, -0.166) ^***^ | -0.191 (-0.216, -0.166) ^***^ | -0.157 (-0.183, -0.132) ^***^ |
| ≥6 (largest figures) | 1523 (16.37) | -0.351 (-0.380, -0.321) ^***^ | -0.351 (-0.380, -0.321) ^***^ | -0.296 (-0.327, -0.266) ^***^ |
| *P*_trend_ |  | <0.001 | <0.001 | <0.001 |
| Per 1-figure increase |  | -0.133 (-0.145, -0.121) ^***^ | -0.133 (-0.145, -0.121) ^***^ | -0.114 (-0.126, -0.101) ^***^ |
| Early adulthood (18-30 years) | | | | |
| ≤3 (smallest figures) | 368 (3.96) | -0.062 (-0.118, -0.006) ^*^ | -0.063 (-0.118, -0.007) ^*^ | -0.022 (-0.076, 0.032) |
| 4 | 3578 (38.46) | 0.000 (ref) | 0.000 (ref) | 0.000 (ref) |
| 5 | 3674 (39.49) | -0.070 (-0.094, -0.046) ^***^ | -0.070 (-0.094, -0.046) ^***^ | -0.047 (-0.071, -0.023) ^***^ |
| ≥6 (largest figures) | 1683 (18.09) | -0.286 (-0.316, -0.256) ^***^ | -0.286 (-0.316, -0.256) ^***^ | -0.220 (-0.250, -0.189) ^***^ |
| *P*_trend_ |  | <0.001 | <0.001 | <0.001 |
| Per 1-figure increase |  | -0.087 (-0.098, -0.077) ^***^ | -0.087 (-0.098, -0.076) ^***^ | -0.072 (-0.083, -0.061) ^***^ |
| Midlife (31-50 years) | | | | |
| ≤3 (smallest figures) | 221 (2.38) | -0.033 (-0.106, 0.040) | -0.035 (-0.108, 0.037) | -0.010 (-0.080, 0.059) |
| 4 | 2629 (28.26) | 0.000 (ref) | 0.000 (ref) | 0.000 (ref) |
| 5 | 3343 (35.93) | -0.049 (-0.076, -0.022) ^***^ | -0.048 (-0.075, -0.021) ^***^ | 0.005 (-0.021, 0.032) |
| ≥6 (largest figures) | 3110 (33.43) | -0.081 (-0.109, -0.054) ^***^ | -0.079 (-0.107, -0.052) ^***^ | -0.009 (-0.037, 0.020) |
| *P*_trend_ |  | <0.001 | <0.001 | <0.001 |
| Per 1-figure increase |  | -0.040 (-0.050, -0.030) ^***^ | -0.039 (-0.050, -0.029) ^***^ | -0.016 (-0.026, -0.005) ^**^ |
| Current | | | | |
| ≤3 (smallest figures) | 188 (2.02) | -0.060 (-0.139, 0.018) | -0.064 (-0.143, 0.014) | -0.038 (-0.113, 0.037) |
| 4 | 2389 (25.68) | 0.000 (ref) | 0.000 (ref) | 0.000 (ref) |
| 5 | 2454 (26.38) | -0.116 (-0.145, -0.086) ^***^ | -0.114 (-0.144, -0.084) ^***^ | -0.024 (-0.053, 0.006) |
| ≥6 (largest figures) | 4272 (45.92) | -0.009 (-0.036, 0.017) | -0.007 (-0.033, 0.020) | 0.056 (0.029, 0.084) ^***^ |
| *P*_trend_ |  | <0.001 | <0.001 | <0.001 |
| Per 1-figure increase |  | 0.001 (-0.008, 0.010) | 0.002 (-0.007, 0.011) | 0.014 (0.005, 0.024) ^**^ |

CI: confidence interval

^†^ Model 1 were adjusted for sex, age.

‡ Model 2 were additionally adjusted for education, occupation, family annual income and childhood socio-economic disadvantage.

^*^*P*<0.05, ^**^*P*<0.01, ^***^*P*<0.001

**Table S3** Association between body size at each life stage with the attention and calculation domain

|  | N (%) | Crude model,  β (95% CI) | Model 1,  β (95% CI) † | Model 2,  β (95% CI) ‡ |
| --- | --- | --- | --- | --- |
| Childhood (6-12 years) | | | | |
| ≤3 (smallest figures) | 1016 (10.92) | -0.213 (-0.286, -0.141) ^***^ | -0.190 (-0.263, -0.118) ^***^ | -0.116 (-0.186, -0.046) ^**^ |
| 4 | 4949 (53.20) | 0.000 (ref) | 0.000 (ref) | 0.000 (ref) |
| 5 | 1849 (19.88) | -0.336 (-0.393, -0.279) ^***^ | -0.347 (-0.404, -0.290) ^***^ | -0.270 (-0.327, -0.213) ^***^ |
| ≥6 (largest figures) | 1489 (16.01) | -0.517 (-0.580, -0.455) ^***^ | -0.530 (-0.593, -0.468) ^***^ | -0.375 (-0.438, -0.313) ^***^ |
| *P*_trend_ |  | <0.001 | <0.001 | <0.001 |
| Per 1-figure increase |  | -0.161 (-0.185, -0.138) ^***^ | -0.172 (-0.196, -0.149) ^***^ | -0.127 (-0.151, -0.103) ^***^ |
| Adolescence (13-17 years) | | | | |
| ≤3 (smallest figures) | 554 (5.96) | -0.301 (-0.396, -0.207) ^***^ | -0.272 (-0.366, -0.177) ^***^ | -0.159 (-0.249, -0.069) ^***^ |
| 4 | 4830 (51.92) | 0.000 (ref) | 0.000 (ref) | 0.000 (ref) |
| 5 | 2396 (25.76) | -0.309 (-0.362, -0.257) ^***^ | -0.321 (-0.373, -0.268) ^***^ | -0.220 (-0.273, -0.168) ^***^ |
| ≥6 (largest figures) | 1523 (16.37) | -0.522 (-0.584, -0.460) ^***^ | -0.536 (-0.598, -0.475) ^***^ | -0.368 (-0.430, -0.305) ^***^ |
| *P*_trend_ |  | <0.001 | <0.001 | <0.001 |
| Per 1-figure increase |  | -0.181 (-0.206, -0.156) ^***^ | -0.192 (-0.217, -0.167) ^***^ | -0.134 (-0.160, -0.109) ^***^ |
| Early adulthood (18-30 years) | | | | |
| ≤3 (smallest figures) | 368 (3.96) | -0.327 (-0.443, -0.212) ^***^ | -0.301 (-0.417, -0.186) ^***^ | -0.169 (-0.280, -0.059) ^**^ |
| 4 | 3578 (38.46) | 0.000 (ref) | 0.000 (ref) | 0.000 (ref) |
| 5 | 3674 (39.49) | -0.220 (-0.270, -0.170) ^***^ | -0.231 (-0.281, -0.182) ^***^ | -0.163 (-0.211, -0.115) ^***^ |
| ≥6 (largest figures) | 1683 (18.09) | -0.473 (-0.535, -0.410) ^***^ | -0.494 (-0.557, -0.432) ^***^ | -0.314 (-0.377, -0.252) ^***^ |
| *P*_trend_ |  | <0.001 | <0.001 | <0.001 |
| Per 1-figure increase |  | -0.133 (-0.156, -0.110) ^***^ | -0.143 (-0.166, -0.120) ^***^ | -0.101 (-0.124, -0.079) ^***^ |
| Midlife (31-50 years) | | | | |
| ≤3 (smallest figures) | 221 (2.38) | -0.355 (-0.504, -0.207) ^***^ | -0.330 (-0.478, -0.182) ^***^ | -0.232 (-0.372, -0.091) ^**^ |
| 4 | 2629 (28.26) | 0.000 (ref) | 0.000 (ref) | 0.000 (ref) |
| 5 | 3343 (35.93) | -0.276 (-0.331, -0.221) ^***^ | -0.288 (-0.343, -0.233) ^***^ | -0.183 (-0.237, -0.129) ^***^ |
| ≥6 (largest figures) | 3110 (33.43) | -0.417 (-0.473, -0.361) ^***^ | -0.437 (-0.493, -0.381) ^***^ | -0.290 (-0.347, -0.233) ^***^ |
| *P*_trend_ |  | <0.001 | <0.001 | <0.001 |
| Per 1-figure increase |  | -0.128 (-0.149, -0.108) ^***^ | -0.137 (-0.158, -0.116) ^***^ | -0.084 (-0.105, -0.064) ^***^ |
| Current | | | | |
| ≤3 (smallest figures) | 188 (2.02) | -0.439 (-0.600, -0.279) ^***^ | -0.407 (-0.567, -0.246) ^***^ | -0.322 (-0.474, -0.170) ^***^ |
| 4 | 2389 (25.68) | 0.000 (ref) | 0.000 (ref) | 0.000 (ref) |
| 5 | 2454 (26.38) | -0.384 (-0.445, -0.323) ^***^ | -0.398 (-0.459, -0.337) ^***^ | -0.214 (-0.273, -0.155) ^***^ |
| ≥6 (largest figures) | 4272 (45.92) | -0.304 (-0.358, -0.250) ^***^ | -0.322 (-0.376, -0.268) ^***^ | -0.212 (-0.267, -0.156) ^***^ |
| *P*_trend_ |  | <0.001 | <0.001 | <0.001 |
| Per 1-figure increase |  | -0.070 (-0.089, -0.051) ^***^ | -0.077 (-0.096, -0.058) ^***^ | -0.053 (-0.073, -0.034) ^***^ |

CI: confidence interval

^†^ Model 1 were adjusted for sex, age.

‡ Model 2 were additionally adjusted for education, occupation, family annual income and childhood socio-economic disadvantage.

^**^*P*<0.01, ^***^*P*<0.001

**Table S4** Association between body size at each life stage with the recall-domain

|  | N (%) | Crude model,  β (95% CI) | Model 1,  β (95% CI) † | Model 2,  β (95% CI) ‡ |
| --- | --- | --- | --- | --- |
| Childhood (6-12 years) | | | | |
| ≤3 (smallest figures) | 1016 (10.92) | -0.209 (-0.261, -0.157) ^***^ | -0.199 (-0.251, -0.147) ^***^ | -0.120 (-0.172, -0.068) ^***^ |
| 4 | 4949 (53.20) | 0.000 (ref) | 0.000 (ref) | 0.000 (ref) |
| 5 | 1849 (19.88) | -0.021 (-0.063, 0.020) | -0.026 (-0.067, 0.015) | -0.045 (-0.087, -0.002) ^*^ |
| ≥6 (largest figures) | 1489 (16.01) | -0.025 (-0.069, 0.020) ^***^ | -0.032 (-0.077, 0.013) | 0.003 (-0.043, 0.050) |
| *P*_trend_ |  | <0.001 | <0.001 | <0.001 |
| Per 1-figure increase |  | 0.030 (0.013, 0.047) ^***^ | 0.025 (0.008, 0.042) ^**^ | 0.017 (-0.001, 0.035) |
| Adolescence (13-17 years) | | | | |
| ≤3 (smallest figures) | 554 (5.96) | -0.124 (-0.193, -0.056) ^***^ | -0.108 (-0.176, -0.040) ^**^ | -0.018 (-0.085, 0.050) |
| 4 | 4830 (51.92) | 0.000 (ref) | 0.000 (ref) | 0.000 (ref) |
| 5 | 2396 (25.76) | -0.038 (-0.076, 0.000) ^*^ | -0.041 (-0.079, -0.003) ^*^ | -0.045 (-0.084, -0.006) ^*^ |
| ≥6 (largest figures) | 1523 (16.37) | -0.007 (-0.052, 0.038) | -0.014 (-0.058, 0.031) | 0.019 (-0.028, 0.065) |
| *P*_trend_ |  | <0.001 | <0.001 | <0.001 |
| Per 1-figure increase |  | 0.008 (-0.010, 0.025) | 0.003 (-0.015, 0.020) | 0.000 (-0.019, 0.019) |
| Early adulthood (18-30 years) | | | | |
| ≤3 (smallest figures) | 368 (3.96) | -0.095 (-0.178, -0.011) ^*^ | -0.080 (-0.163, 0.003) | 0.047 (-0.035, 0.129) |
| 4 | 3578 (38.46) | 0.000 (ref) | 0.000 (ref) | 0.000 (ref) |
| 5 | 3674 (39.49) | -0.083 (-0.118, -0.047) ^***^ | -0.086 (-0.122, -0.051) ^***^ | -0.050 (-0.086, -0.014) ^**^ |
| ≥6 (largest figures) | 1683 (18.09) | -0.032 (-0.077, 0.013) | -0.042 (-0.087, 0.003) | 0.013 (-0.034, 0.059) |
| *P*_trend_ |  | <0.001 | <0.001 | <0.001 |
| Per 1-figure increase |  | -0.002 (-0.019, 0.014) | -0.007 (-0.023, 0.009) | -0.004 (-0.021, 0.013) |
| Midlife (31-50 years) | | | | |
| ≤3 (smallest figures) | 221 (2.38) | -0.182 (-0.288, -0.077) ^***^ | -0.168 (-0.274, -0.062) ^**^ | -0.038 (-0.143, 0.066) |
| 4 | 2629 (28.26) | 0.000 (ref) | 0.000 (ref) | 0.000 (ref) |
| 5 | 3343 (35.93) | -0.213 (-0.253, -0.174) ^***^ | -0.223 (-0.263, -0.184) ^***^ | -0.177 (-0.217, -0.137) ^***^ |
| ≥6 (largest figures) | 3110 (33.43) | -0.164 (-0.204, -0.124) ^***^ | -0.174 (-0.214, -0.134) ^***^ | -0.093 (-0.135, -0.051) ^***^ |
| *P*_trend_ |  | <0.001 | <0.001 | <0.001 |
| Per 1-figure increase |  | -0.025 (-0.040, -0.011) ^***^ | -0.030 (-0.045, -0.015) ^***^ | -0.007 (-0.023, 0.008) |
| Current | | | | |
| ≤3 (smallest figures) | 188 (2.02) | -0.146 (-0.260, -0.031) ^*^ | -0.122 (-0.236, -0.008) ^*^ | -0.013 (-0.125, 0.099) |
| 4 | 2389 (25.68) | 0.000 (ref) | 0.000 (ref) | 0.000 (ref) |
| 5 | 2454 (26.38) | -0.220 (-0.263, -0.176) ^***^ | -0.229 (-0.273, -0.186) ^***^ | -0.192 (-0.236, -0.148) ^***^ |
| ≥6 (largest figures) | 4272 (45.92) | -0.193 (-0.231, -0.154) ^***^ | -0.204 (-0.242, -0.165) ^***^ | -0.129 (-0.170, -0.088) ^***^ |
| *P*_trend_ |  | <0.001 | <0.001 | <0.001 |
| Per 1-figure increase |  | -0.048 (-0.061, -0.034) ^***^ | -0.052 (-0.065, -0.038) ^***^ | -0.030 (-0.044, -0.015) ^***^ |

CI: confidence interval

^†^ Model 1 were adjusted for sex, age.

‡ Model 2 were additionally adjusted for education, occupation, family annual income and childhood socio-economic disadvantage.

^*^*P*<0.05, ^**^*P*<0.01, ^***^*P*<0.001

**Table S5** Association between body size at each life stage with the language and praxis domain

|  | N (%) | Crude model,  β (95% CI) | Model 1,  β (95% CI) † | Model 2,  β (95% CI) ‡ |
| --- | --- | --- | --- | --- |
| Childhood (6-12 years) | | | | |
| ≤3 (smallest figures) | 1016 (10.92) | 0.088 (0.029, 0.147) ^**^ | 0.087 (0.028, 0.147) ^**^ | 0.120 (0.065, 0.176) ^***^ |
| 4 | 4949 (53.20) | 0.000 (ref) | 0.000 (ref) | 0.000 (ref) |
| 5 | 1849 (19.88) | -0.586 (-0.633, -0.539) ^***^ | -0.586 (-0.633, -0.539) ^***^ | -0.451 (-0.497, -0.406) ^***^ |
| ≥6 (largest figures) | 1489 (16.01) | -0.716 (-0.766, -0.665) | -0.714 (-0.765, -0.663) ^***^ | -0.525 (-0.574, -0.476) ^***^ |
| *P*_trend_ |  | <0.001 | <0.001 | <0.001 |
| Per 1-figure increase |  | -0.310 (-0.329, -0.291) ^***^ | -0.310 (-0.329, -0.291) ^***^ | -0.236 (-0.255, -0.217) ^***^ |
| Adolescence (13-17 years) | | | | |
| ≤3 (smallest figures) | 554 (5.96) | 0.056 (-0.021, 0.134) | 0.053 (-0.025, 0.131) | 0.130 (0.058, 0.202) ^***^ |
| 4 | 4830 (51.92) | 0.000 (ref) | 0.000 (ref) | 0.000 (ref) |
| 5 | 2396 (25.76) | -0.483 (-0.526, -0.440) ^***^ | -0.485 (-0.529, -0.442) ^***^ | -0.327 (-0.369, -0.286) ^***^ |
| ≥6 (largest figures) | 1523 (16.37) | -0.711 (-0.762, -0.661) ^***^ | -0.711 (-0.762, -0.660) ^***^ | -0.496 (-0.546, -0.446) ^***^ |
| *P*_trend_ |  | <0.001 | <0.001 | <0.001 |
| Per 1-figure increase |  | -0.313 (-0.334, -0.293) ^***^ | -0.313 (-0.334, -0.293) ^***^ | -0.226 (-0.246, -0.206) ^***^ |
| Early adulthood (18-30 years) | | | | |
| ≤3 (smallest figures) | 368 (3.96) | 0.089 (-0.008, 0.185) | 0.086 (-0.011, 0.182) | 0.147 (0.058, 0.236) ^**^ |
| 4 | 3578 (38.46) | 0.000 (ref) | 0.000 (ref) | 0.000 (ref) |
| 5 | 3674 (39.49) | -0.259 (-0.301, -0.218) ^***^ | -0.261 (-0.302, -0.219) ^***^ | -0.207 (-0.246, -0.168) ^***^ |
| ≥6 (largest figures) | 1683 (18.09) | -0.645 (-0.697, -0.593) ^***^ | -0.645 (-0.697, -0.592) ^***^ | -0.440 (-0.490, -0.389) ^***^ |
| *P*_trend_ |  | <0.001 | <0.001 | <0.001 |
| Per 1-figure increase |  | -0.222 (-0.241, -0.203) ^***^ | -0.221 (-0.240, -0.202) ^***^ | -0.164 (-0.182, -0.145) ^***^ |
| Midlife (31-50 years) | | | | |
| ≤3 (smallest figures) | 221 (2.38) | 0.059 (-0.067, 0.185) | 0.053 (-0.073, 0.179) | 0.052 (-0.063, 0.166) |
| 4 | 2629 (28.26) | 0.000 (ref) | 0.000 (ref) | 0.000 (ref) |
| 5 | 3343 (35.93) | -0.273 (-0.320, -0.226) ^***^ | -0.268 (-0.315, -0.221) ^***^ | -0.172 (-0.216, -0.128) ^***^ |
| ≥6 (largest figures) | 3110 (33.43) | -0.340 (-0.388, -0.292) ^***^ | -0.337 (-0.385, -0.289) ^***^ | -0.207 (-0.253, -0.160) ^***^ |
| *P*_trend_ |  | <0.001 | <0.001 | <0.001 |
| Per 1-figure increase |  | -0.137 (-0.154, -0.119) ^***^ | -0.135 (-0.153, -0.118) ^***^ | -0.080 (-0.097, -0.063) ^***^ |
| Current | | | | |
| ≤3 (smallest figures) | 188 (2.02) | 0.029 (-0.107, 0.164) | 0.017 (-0.119, 0.153) | 0.019 (-0.105, 0.142) |
| 4 | 2389 (25.68) | 0.000 (ref) | 0.000 (ref) | 0.000 (ref) |
| 5 | 2454 (26.38) | -0.462 (-0.513, -0.410) ^***^ | -0.457 (-0.508, -0.405) ^***^ | -0.238 (-0.286, -0.190) ^***^ |
| ≥6 (largest figures) | 4272 (45.92) | -0.203 (-0.248, -0.157) ^***^ | -0.198 (-0.244, -0.152) ^***^ | -0.121 (-0.167, -0.076) ^***^ |
| *P*_trend_ |  | <0.001 | <0.001 | <0.001 |
| Per 1-figure increase |  | -0.046 (-0.062, -0.030) ^***^ | -0.043 (-0.060, -0.027) ^***^ | -0.037 (-0.053, -0.022) ^***^ |

CI: confidence interval

^†^ Model 1 were adjusted for sex, age.

‡ Model 2 were additionally adjusted for education, occupation, family annual income and childhood socio-economic disadvantage.

^**^*P*<0.01, ^***^*P*<0.001

**Table S6** Sex-specific posterior mean weights of body size across life stages and their 95% credible intervals on midlife and late-life cognitive function

|  | Relative weight (%), mean (95% CrI) ^a^ |
| --- | --- |
| **Women (n = 6,656)** |  |
| Childhood (6-12 years) | 55.1 (44.41, 65.96) |
| Adolescence (13-17 years) | 41.6 (30.43, 52.51) |
| Early adulthood (18-30 years) | 2.28 (0.07, 7.36) |
| Midlife (31-50 years) | 0.67 (0.02, 2.43) |
| Current status (age at baseline, ≥50 years) | 0.34 (0.01, 1.25) |
| **Men (n = 2,647)** |  |
| Childhood (6-12 years) | 67.46 (50.32, 84.73) |
| Adolescence (13-17 years) | 26.37 (8.5, 44.08) |
| Early adulthood (18-30 years) | 4.4 (0.14, 13.4) |
| Midlife (31-50 years) | 1.2 (0.03, 4.29) |
| Current status (age at baseline, ≥50 years) | 0.58 (0.01, 2.1) |

CrI: credible interval

^a^ The BRLM model for estimating relative weights across life stages adjusted for age, education, occupation, family annual income and childhood socio-economic disadvantage (CSD).

**Table S7** Age-specific posterior mean weights of body size across life stages and their 95% credible intervals on midlife and late-life cognitive function

|  | Relative weight (%), mean (95% CrI) ^a^ |
| --- | --- |
| **Age < 65 (n = 7,687)** |  |
| Childhood (6-12 years) | 57.83 (48.37, 67.46) |
| Adolescence (13-17 years) | 39.86 (30.04, 49.43) |
| Early adulthood (18-30 years) | 1.58 (0.05, 5.35) |
| Midlife (31-50 years) | 0.48 (0.01, 1.76) |
| Current status (age at baseline, ≥50 years) | 0.24 (0.01, 0.87) |
| **Age ≥ 65 (n = 1,616)** |  |
| Childhood (6-12 years) | 56.63 (27.07, 82.99) |
| Adolescence (13-17 years) | 25.04 (2.1, 54.54) |
| Early adulthood (18-30 years) | 13.44 (0.64, 34.92) |
| Midlife (31-50 years) | 3.05 (0.08, 10.69) |
| Current status (age at baseline, ≥50 years) | 1.86 (0.05, 6.69) |

CrI: credible interval

^a^ The BRLM model for estimating relative weights across life stages adjusted for sex, education, occupation, family annual income and childhood socio-economic disadvantage (CSD).

**Table S8** Association between body size at each life stage and MMSE scores further adjusting for current body mass index

|  | N (%) | Model 1,  β (95% CI) † | Model 2,  β (95% CI) ‡ | Model 3,  β (95% CI) § |
| --- | --- | --- | --- | --- |
| Childhood (6-12 years) | | | | |
| ≤3 (smallest figures) | 1007 (11.0) | -0.232 (-0.478, 0.013) | -0.193 (-0.439, 0.054) | 0.145 (-0.082, 0.371) |
| 4 | 4918 (53.5) | 0.000 (ref) | 0.000 (ref) | 0.000 (ref) |
| 5 | 1812 (19.7) | -2.411 (-2.606, -2.216) ^***^ | -2.430 (-2.625, -2.235) ^***^ | -2.028 (-2.214, -1.841) ^***^ |
| ≥6 (largest figures) | 1460 (15.9) | -3.541 (-3.752, -3.330) ^*^ | -3.560 (-3.771, -3.348) ^***^ | -2.777 (-2.980, -2.573) ^***^ |
| *P*_trend_ |  | <0.001 | <0.001 | <0.001 |
| Per 1-figure increase |  | -1.349 (-1.429, -1.268) ^***^ | -1.369 (-1.450, -1.287) ^***^ | -1.125 (-1.204, -1.046) ^***^ |
| Adolescence (13-17 years) | | | | |
| ≤3 (smallest figures) | 549 (6.0) | -0.363 (-0.686, -0.040) ^*^ | -0.319 (-0.643, 0.004) | 0.150 (-0.147, 0.447) |
| 4 | 4804 (52.2) | 0.000 (ref) | 0.000 (ref) | 0.000 (ref) |
| 5 | 2352 (25.6) | -1.980 (-2.161, -1.799) ^***^ | -2.005 (-2.186, -1.823) ^***^ | -1.455 (-1.628, -1.281) ^***^ |
| ≥6 (largest figures) | 1492 (16.2) | -3.452 (-3.664, -3.240) ^***^ | -3.475 (-3.688, -3.262) ^***^ | -2.592 (-2.799, -2.386) ^***^ |
| *P*_trend_ |  | <0.001 | <0.001 | <0.001 |
| Per 1-figure increase |  | -1.382 (-1.468, -1.296) ^***^ | -1.402 (-1.489, -1.316) ^***^ | -1.081 (-1.166, -0.997) ^***^ |
| Early adulthood (18-30 years) | | | | |
| ≤3 (smallest figures) | 363 (4.0) | -0.235 (-0.637, 0.167) | -0.196 (-0.598, 0.207) | 0.315 (-0.052, 0.683) |
| 4 | 3554 (38.6) | 0.000 (ref) | 0.000 (ref) | 0.000 (ref) |
| 5 | 3626 (39.4) | -1.181 (-1.355, -1.008) ^***^ | -1.205 (-1.379, -1.031) ^***^ | -0.879 (-1.040, -0.718) ^***^ |
| ≥6 (largest figures) | 1654 (18.0) | -3.154 (-3.372, -2.936) ^***^ | -3.192 (-3.411, -2.973) ^***^ | -2.241 (-2.451, -2.031) ^***^ |
| *P*_trend_ |  | <0.001 | <0.001 | <0.001 |
| Per 1-figure increase |  | -1.032 (-1.111, -0.952) ^***^ | -1.048 (-1.128, -0.968) ^***^ | -0.803 (-0.879, -0.726) ^***^ |
| Midlife (31-50 years) | | | | |
| ≤3 (smallest figures) | 218 (2.4) | -0.341 (-0.868, 0.185) | -0.313 (-0.841, 0.214) | -0.044 (-0.521, 0.433) |
| 4 | 2608 (28.4) | 0.000 (ref) | 0.000 (ref) | 0.000 (ref) |
| 5 | 3303 (35.9) | -1.253 (-1.448, -1.057) ^***^ | -1.262 (-1.458, -1.066) ^***^ | -0.739 (-0.920, -0.557) ^***^ |
| ≥6 (largest figures) | 3068 (33.4) | -1.964 (-2.164, -1.763) ^***^ | -1.989 (-2.190, -1.787) ^***^ | -1.208 (-1.403, -1.013) ^***^ |
| *P*_trend_ |  | <0.001 | <0.001 | <0.001 |
| Per 1-figure increase |  | -0.745 (-0.818, -0.671) ^***^ | -0.755 (-0.829, -0.681) ^***^ | -0.465 (-0.536, -0.394) ^***^ |
| Current status (age at baseline, aged ≥50 years) | | | | |
| ≤3 (smallest figures) | 185 (2.0) | -0.530 (-1.103, 0.043) | -0.506 (-1.080, 0.068) | -0.263 (-0.780, 0.254) |
| 4 | 2359 (25.7) | 0.000 (ref) | 0.000 (ref) | 0.000 (ref) |
| 5 | 2421 (26.3) | -1.929 (-2.145, -1.712) ^***^ | -1.940 (-2.157, -1.723) ^***^ | -0.968 (-1.168, -0.768) ^***^ |
| ≥6 (largest figures) | 4232 (46.0) | -1.309 (-1.502, -1.116) ^***^ | -1.324 (-1.518, -1.130) ^***^ | -0.759 (-0.949, -0.570) ^***^ |
| *P*_trend_ |  | <0.001 | <0.001 | <0.001 |
| Per 1-figure increase |  | -0.373 (-0.442, -0.305) ^***^ | -0.378 (-0.447, -0.309) ^***^ | -0.267 (-0.334, -0.201) ^***^ |

CI: confidence interval

^†^ Model 1 were adjusted for current body mass index.

‡ Model 2 were additionally adjusted for sex, age.

§ Model 3 were additionally adjusted for education, occupation, family annual income and childhood socio-economic disadvantage (CSD).

^*^*P*<0.05, ^***^*P*<0.001

**Table S9** Posterior mean weights of body size across life stages and their 95% credible intervals on midlife and late-life cognitive function further adjusting for current body mass index

|  | Relative weight (%), mean (95% CrI) ^a^ |
| --- | --- |
| Childhood (6-12 years) | 58.56 (49.49, 67.68) |
| Adolescence (13-17 years) | 38.63 (29.19, 47.83) |
| Early adulthood (18-30 years) | 2.06 (0.06, 6.54) |
| Midlife (31-50 years) | 0.51 (0.01, 1.86) |
| Current status (age at baseline, ≥50 years) | 0.24 (0.01, 0.87) |

CrI: credible interval

^a^ The BRLM model for estimating relative weights across life stages adjusted for sex, age, education, occupation, family annual income, childhood socio-economic disadvantage (CSD), and current body mass index.

**Table S10** Sex-specific posterior mean weights of body size across life stages and their 95% credible intervals on midlife and late-life cognitive function further adjusting for current body mass index

|  | Relative weight (%), mean (95% CrI) ^a^ |
| --- | --- |
| **Women (n = 6,580)** |  |
| Childhood (6-12 years) | 55.43 (44.79, 66.25) |
| Adolescence (13-17 years) | 40.87 (29.58, 51.85) |
| Early adulthood (18-30 years) | 2.57 (0.08, 8.2) |
| Midlife (31-50 years) | 0.75 (0.02, 2.69) |
| Current status (age at baseline, ≥50 years) | 0.37 (0.01, 1.34) |
| **Men (n = 2,617)** |  |
| Childhood (6-12 years) | 65.5 (48.52, 82.24) |
| Adolescence (13-17 years) | 28.33 (10.75, 46.15) |
| Early adulthood (18-30 years) | 4.28 (0.17, 13.08) |
| Midlife (31-50 years) | 1.3 (0.03, 4.73) |
| Current status (age at baseline, ≥50 years) | 0.59 (0.01, 2.17) |

CrI: credible interval

^a^ The BRLM model for estimating relative weights across life stages adjusted for age, education, occupation, family annual income, childhood socio-economic disadvantage (CSD), and current body mass index.

**Table S11** Age-specific posterior mean weights of body size across life stages and their 95% credible intervals on midlife and late-life cognitive function further adjusting for current body mass index

|  | Relative weight (%), mean (95% CrI) ^a^ |
| --- | --- |
| **Age < 65 (n = 7,603)** |  |
| Childhood (6-12 years) | 57.39 (47.66, 67.27) |
| Adolescence (13-17 years) | 40.09 (30.08, 49.92) |
| Early adulthood (18-30 years) | 1.74 (0.05, 5.75) |
| Midlife (31-50 years) | 0.53 (0.01, 1.91) |
| Current status (age at baseline, ≥50 years) | 0.26 (0.01, 0.93) |
| **Age ≥ 65 (n = 1,594)** |  |
| Childhood (6-12 years) | 56.23 (27.51, 81.68) |
| Adolescence (13-17 years) | 25.22 (2.33, 53.8) |
| Early adulthood (18-30 years) | 12.91 (0.59, 33.25) |
| Midlife (31-50 years) | 3.67 (0.11, 12.74) |
| Current status (age at baseline, ≥50 years) | 1.98 (0.06, 7.12) |

CrI: credible interval

^a^ The BRLM model for estimating relative weights across life stages adjusted for sex, education, occupation, family annual income, childhood socio-economic disadvantage (CSD), and current body mass index.

**Table S12** Association between body size at each life stage and MMSE scores after excluding those with an MMSE recall-domain score < 2

|  | N (%) | Crude model,  β (95% CI) | Model 1,  β (95% CI) † | Model 2,  β (95% CI) ‡ |
| --- | --- | --- | --- | --- |
| Childhood (6-12 years) | | | | |
| ≤3 (smallest figures) | 848 (10.5) | -0.307 (-0.522, -0.091) ^**^ | -0.276 (-0.492, -0.060) ^*^ | -0.043 (-0.242, 0.157) |
| 4 | 4336 (53.5) | 0.000 (ref) | 0.000 (ref) | 0.000 (ref) |
| 5 | 1579 (19.5) | -1.558 (-1.726, -1.389) ^***^ | -1.571 (-1.739, -1.402) ^***^ | -1.349 (-1.510, -1.189) ^***^ |
| ≥6 (largest figures) | 1336 (16.5) | -3.252 (-3.432, -3.072) ^***^ | -3.263 (-3.443, -3.083) ^***^ | -2.707 (-2.880, -2.534) ^***^ |
| *P*_trend_ |  | <0.001 | <0.001 | <0.001 |
| Per 1-figure increase |  | -1.131 (-1.200, -1.062) ^***^ | -1.146 (-1.216, -1.077) ^***^ | -0.983 (-1.051, -0.916) ^***^ |
| Adolescence (13-17 years) | | | | |
| ≤3 (smallest figures) | 493 (6.1) | -0.634 (-0.909, -0.359) ^***^ | -0.601 (-0.876, -0.325) ^***^ | -0.300 (-0.554, -0.045) ^*^ |
| 4 | 4184 (51.7) | 0.000 (ref) | 0.000 (ref) | 0.000 (ref) |
| 5 | 2055 (25.4) | -1.390 (-1.546, -1.235) ^***^ | -1.415 (-1.571, -1.260) ^***^ | -1.043 (-1.192, -0.894) ^***^ |
| ≥6 (largest figures) | 1367 (16.9) | -3.205 (-3.384, -3.025) ^***^ | -3.223 (-3.403, -3.043) ^***^ | -2.599 (-2.773, -2.424) ^***^ |
| *P*_trend_ |  | <0.001 | <0.001 | <0.001 |
| Per 1-figure increase |  | -1.169 (-1.242, -1.096) ^***^ | -1.188 (-1.261, -1.115) ^***^ | -0.962 (-1.034, -0.890) ^***^ |
| Early adulthood (18-30 years) | | | | |
| ≤3 (smallest figures) | 339 (4.2) | -0.569 (-0.905, -0.233) ^***^ | -0.535 (-0.872, -0.199) ^**^ | -0.290 (-0.599, 0.020) |
| 4 | 3143 (38.8) | 0.000 (ref) | 0.000 (ref) | 0.000 (ref) |
| 5 | 3103 (38.3) | -0.764 (-0.913, -0.616) ^***^ | -0.789 (-0.938, -0.640) ^***^ | -0.579 (-0.719, -0.439) ^***^ |
| ≥6 (largest figures) | 1514 (18.7) | -2.855 (-3.039, -2.671) ^***^ | -2.887 (-3.072, -2.703) ^***^ | -2.232 (-2.410, -2.053) ^***^ |
| *P*_trend_ |  | <0.001 | <0.001 | <0.001 |
| Per 1-figure increase |  | -0.905 (-0.972, -0.839) ^***^ | -0.921 (-0.988, -0.854) ^***^ | -0.749 (-0.813, -0.684) ^***^ |
| Midlife (31-50 years) | | | | |
| ≤3 (smallest figures) | 199 (2.5) | -0.707 (-1.151, -0.263) ^**^ | -0.674 (-1.118, -0.229) ^**^ | -0.555 (-0.961, -0.149) ^**^ |
| 4 | 2367 (29.2) | 0.000 (ref) | 0.000 (ref) | 0.000 (ref) |
| 5 | 2802 (34.6) | -0.779 (-0.947, -0.611) ^***^ | -0.783 (-0.951, -0.615) ^***^ | -0.385 (-0.542, -0.227) ^***^ |
| ≥6 (largest figures) | 2731 (33.7) | -1.953 (-2.122, -1.784) ^***^ | -1.985 (-2.155, -1.816) ^***^ | -1.368 (-1.535, -1.201) ^***^ |
| *P*_trend_ |  | <0.001 | <0.001 | <0.001 |
| Per 1-figure increase |  | -0.764 (-0.825, -0.703) ^***^ | -0.777 (-0.838, -0.716) ^***^ | -0.555 (-0.615, -0.495) ^***^ |
| Current status (age at baseline, aged ≥50 years) | | | | |
| ≤3 (smallest figures) | 168 (2.1) | -0.691 (-1.180, -0.202) ^**^ | -0.669 (-1.159, -0.180) ^**^ | -0.565 (-1.009, -0.121) ^*^ |
| 4 | 2160 (26.7) | 0.000 (ref) | 0.000 (ref) | 0.000 (ref) |
| 5 | 2087 (25.8) | -1.189 (-1.376, -1.001) ^***^ | -1.198 (-1.385, -1.010) ^***^ | -0.467 (-0.641, -0.293) ^***^ |
| ≥6 (largest figures) | 3684 (45.5) | -1.383 (-1.548, -1.217) ^***^ | -1.404 (-1.570, -1.238) ^***^ | -0.916 (-1.080, -0.753) ^***^ |
| *P*_trend_ |  | <0.001 | <0.001 | <0.001 |
| Per 1-figure increase |  | -0.483 (-0.541, -0.426) ^***^ | -0.493 (-0.551, -0.435) ^***^ | -0.381 (-0.437, -0.324) ^***^ |

CI: confidence interval

^†^ Model 1 were adjusted for sex, age.

‡ Model 2 were additionally adjusted for education, occupation, family annual income and childhood socio-economic disadvantage (CSD).

^*^*P*<0.05, ^**^*P*<0.01, ^***^*P*<0.001

**Table S13** Posterior mean weights of body size across life stages and their 95% credible intervals on midlife and late-life cognitive function after excluding those with an MMSE recall-domain score < 2

|  | Relative weight (%), mean (95% CrI) ^a^ |
| --- | --- |
| Childhood (6-12 years) | 53.06 (42.8, 63.24) |
| Adolescence (13-17 years) | 40.01 (28.89, 50.76) |
| Early adulthood (18-30 years) | 4.06 (0.21, 10.91) |
| Midlife (31-50 years) | 2.43 (0.08, 7.37) |
| Current status (age at baseline, ≥50 years) | 0.44 (0.01, 1.64) |

CrI: credible interval

^a^ The BRLM model for estimating relative weights across life stages adjusted for sex, age, education, occupation, family annual income and childhood socio-economic disadvantage (CSD).

**Table S14** Sex-specific posterior mean weights of body size across life stages and their 95% credible intervals on midlife and late-life cognitive function after excluding those with an MMSE recall-domain score < 2

|  | Relative weight (%), mean (95% CrI) ^a^ |
| --- | --- |
| **Women (n = 5,797)** |  |
| Childhood (6-12 years) | 47.81 (35.99, 59.63) |
| Adolescence (13-17 years) | 44.62 (31.88, 57.1) |
| Early adulthood (18-30 years) | 4.23 (0.17, 11.43) |
| Midlife (31-50 years) | 2.78 (0.1, 8.45) |
| Current status (age at baseline, ≥50 years) | 0.57 (0.01, 2.04) |
| **Men (n = 2,302)** |  |
| Childhood (6-12 years) | 64.38 (46.35, 81.96) |
| Adolescence (13-17 years) | 22.46 (4.21, 42.15) |
| Early adulthood (18-30 years) | 7.22 (0.31, 19.92) |
| Midlife (31-50 years) | 4.56 (0.15, 13.37) |
| Current status (age at baseline, ≥50 years) | 1.38 (0.04, 4.88) |

CrI: credible interval

^a^ The BRLM model for estimating relative weights across life stages adjusted for age, education, occupation, family annual income and childhood socio-economic disadvantage (CSD).

**Table S15** Age-specific posterior mean weights of body size across life stages and their 95% credible intervals on midlife and late-life cognitive function after excluding those with an MMSE recall-domain score < 2

|  | Relative weight (%), mean (95% CrI) ^a^ |
| --- | --- |
| **Age < 65 (n = 6,743)** |  |
| Childhood (6-12 years) | 49.73 (39.48, 60.09) |
| Adolescence (13-17 years) | 43.81 (32.29, 54.98) |
| Early adulthood (18-30 years) | 3.22 (0.12, 9.29) |
| Midlife (31-50 years) | 2.75 (0.1, 7.93) |
| Current status (age at baseline, ≥50 years) | 0.48 (0.01, 1.72) |
| **Age ≥ 65 (n = 1,356)** |  |
| Childhood (6-12 years) | 61.83 (32.56, 85.57) |
| Adolescence (13-17 years) | 16.29 (0.6, 44.6) |
| Early adulthood (18-30 years) | 14.12 (0.69, 36.43) |
| Midlife (31-50 years) | 4.91 (0.14, 16.64) |
| Current status (age at baseline, ≥50 years) | 2.84 (0.08, 9.82) |

CrI: credible interval

^a^ The BRLM model for estimating relative weights across life stages adjusted for sex, education, occupation, family annual income and childhood socio-economic disadvantage (CSD).
